# Supplementary figures and images for: Spikeling: A low-cost hardware implementation of a spiking neuron for neuroscience teaching and outreach
Source: PLoS Biol. 2018 Oct 26;16(10):e2006760. doi: 10.1371/journal.pbio.2006760 (PMC6221365; doi:10.1371/journal.pbio.2006760)

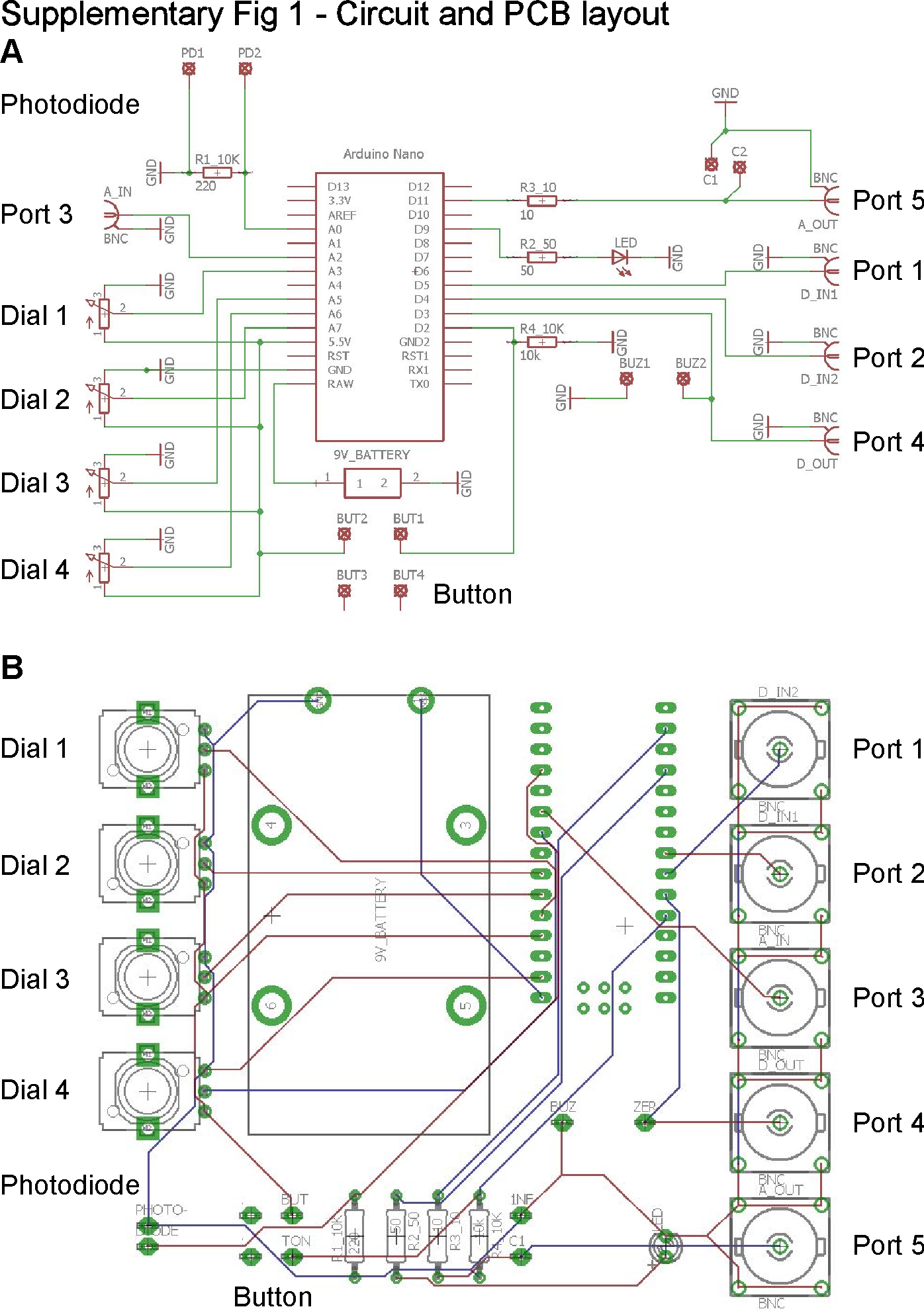

Supplement: S1 Fig — A. Wiring diagram of Spikeling. B. PCB layout. (TIF) [file pbio.2006760.s001.tif]

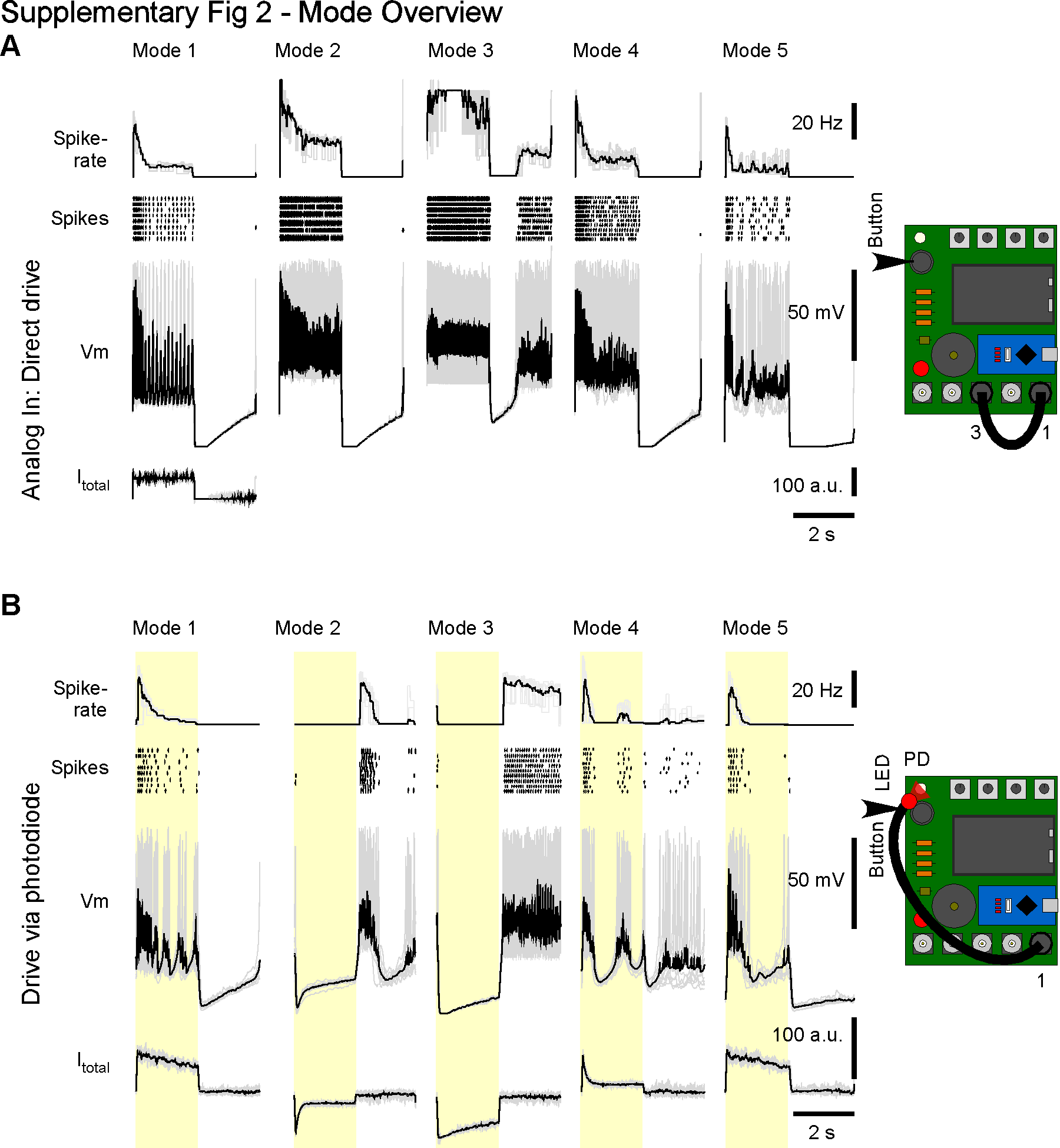

Supplement: S2 Fig — A, B. All 5 preprogrammed Spikeling modes responding to current (A) and light steps (B). Additional modes can be easily added in the Arduino code (see Spikeling manual). (TIF) [file pbio.2006760.s002.tif]
