# Supplementary material for: Spikeling: A low-cost hardware implementation of a spiking neuron for neuroscience teaching and outreach
Source: PLoS Biol. 2018 Oct 26;16(10):e2006760. doi: 10.1371/journal.pbio.2006760 (PMC6221365; doi:10.1371/journal.pbio.2006760)
Supplement: S1 Text — BOM, Bill of Materials; CSV, comma separated variable; LED, light-emitting diode; PCB; STL, surface-tessilation. (PDF) [file pbio.2006760.s007.pdf]

# Spikeling User Manual

## Content

**Assembly From Parts** .......... 2
**Getting Started** .......... 4
**Visualising Spikeling Output with MATLAB** .......... 7
**Visualising Spikeling Output with Python** .......... 10
  **Getting started with Python** .......... 10
  **Using the Juypter Notebook** .......... 11
  **Plotting Stimulus aligned data** .......... 13
  **Plotting STAs** .......... 13
**Reprogramming Spikeling (Arduino)** .......... 14
  **Arduino Implementation of Izhikevich Model** .......... 15
  **Photodiode Adaptation Speed** .......... 17
  **Model Noise** .......... 18
  **Spikeling Refresh Rate** .......... 18
  **Speeding up the model** .......... 18
**Spikeling 2.0** .......... 19
**Spikeling Exercises** .......... 22
  **Resting membrane potential** .......... 22
  **External Stimulation: Light** .......... 23
**Switching spike-modes** .......... 25
  **The "Self-Stimulator"** .......... 27
  **Noise** .......... 31
  **Building networks** .......... 34

*Version 1.  6th Sept 2018.*

## Assembly From Parts

If desired, all parts can be conveniently sourced through Kitspace: https://kitspace.org/boards/github.com/badenlab/spikeling/

When using this option, please be aware:

1) Kitspace will almost certainly not find the cheapest Arduino Nano (clone). You should be able to get one for less than £3. Similarly, PCB manufacturing options suggested may also not be the cheapest. Google is your friend here!
2) Kitspace will likely find some options where “packs” of components are listed (e.g. instead of the 2 optional screws to hold the optional battery pack in place, it may find a box of 100). So, when using the option, do carefully go through the suggested purchase list and edit as appropriate.
3) Obviously, when buying in bulk, things can get a lot cheaper (potentially several-fold savings per unit).

Solder all parts in place as per the below. An introduction to soldering is here: https://www.howtogeek.com/63630/how-to-use-a-soldering-iron-a-beginners-guide//.

For details on parts, see Bill of materials (BOM).

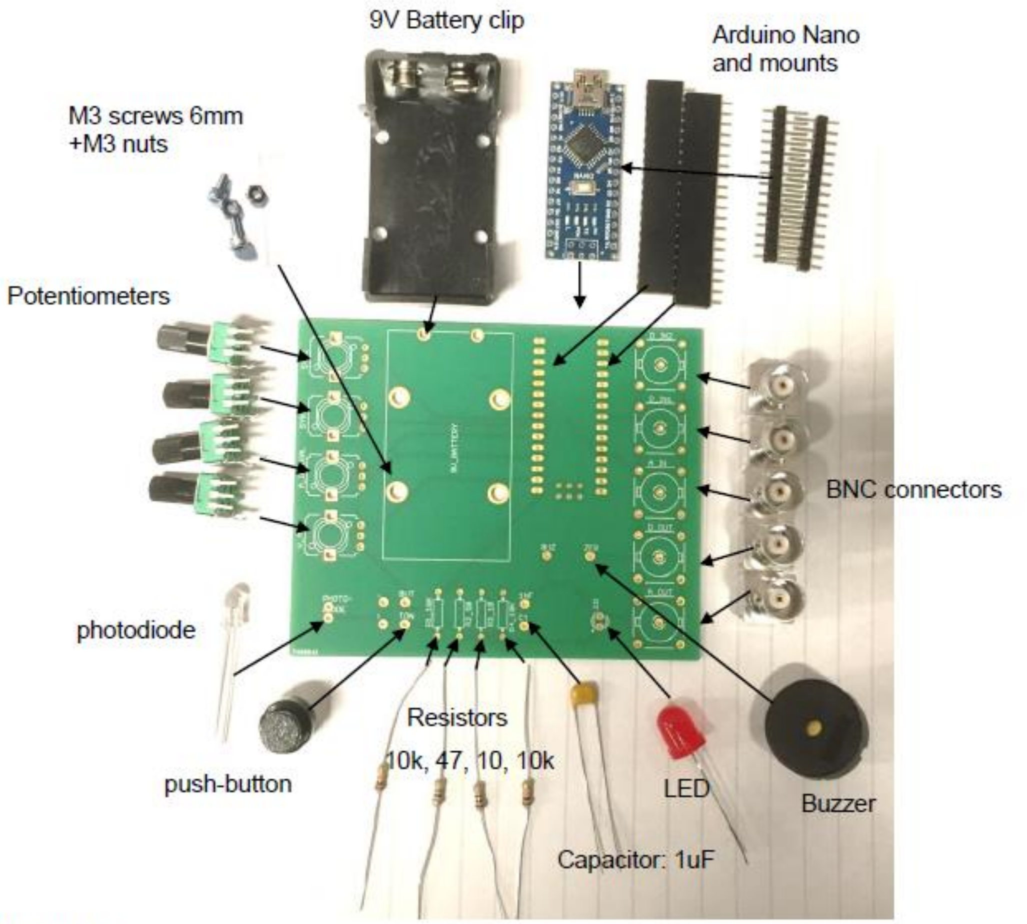


Oriented pieces:
- Photodiode and LED: orient long legs towards the bottom edge of the board
- Button: the two rightmost holes matter, make sure the button isolates them until pressed

When complete, it should hopefully look like this:

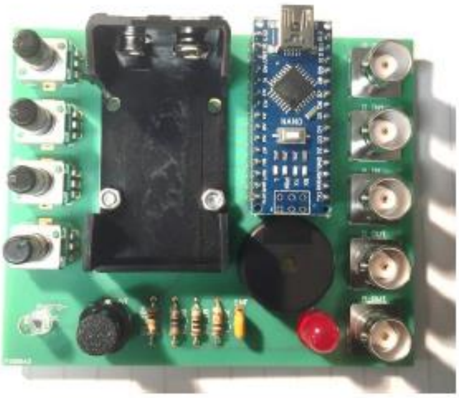

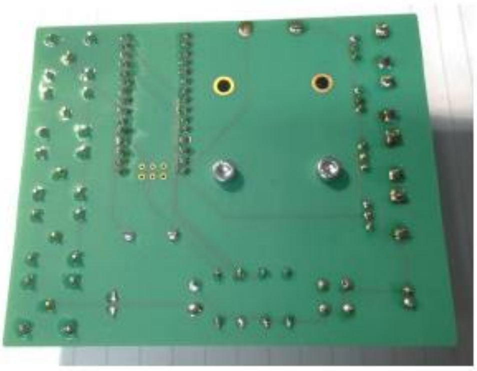

Note: If it doesn't seem to work, 9 out of 10 times it's because 1 of the following:

1) Did you upload the Arduino script? If not sure how to do that, see below under getting started.
2) Somewhere, 2 or more contacts that shouldn't touch, so in fact touch and thus short-circuit something. Carefully check the board (both sides, solder can leak through the holes and make unwanted contacts on the other side). To check, it can be instructive to "beep" the circuit with a multimeter (google that if unsure). If you have a short circuit, remove it. Usually, simply reheating the pad may do the trick, but if not, used a solder sucker.
3) "Cold contacts", i.e. an intended contact that are not actually connected. This can easily happen e.g. if the parts are slightly moved during soldering, such that in the last second, as the solder hardens, the pieces come apart. They may then "look" like they are fine, but don't conduct. Check with multimeter, or simply re-heat all the dodgy looking contacts.
4) Did you solder the oriented pieces the right way round? (see previous page)

*You will know if it works if you power it (Battery or USB cable), with all dials turned to mid-point, if you then crank up dial 4 or hold it into the light, it should start to spike (audible clicks coupled to flashes from the LED). Congratulations, you have successfully built a Spikeling!*

## Getting Started

Turn all dials to midpoint (you should feel a gentle "click") and power the board (via USB, or via 9V Battery). The Arduino LEDs should come on. If the board is already on, press the small white reset button on the Arduino itself (not the big black button on the board). This will take a few seconds and puts Spikeling in "reset state" (i.e. mode 1, with all dials to midpoint). All instructions below assume you start from here.

*The **reset state** mimics the "average textbook neuron" - a vertebrate spiking neuron with a resting membrane potential around -70 mV and a spike threshold around -55 mV.*

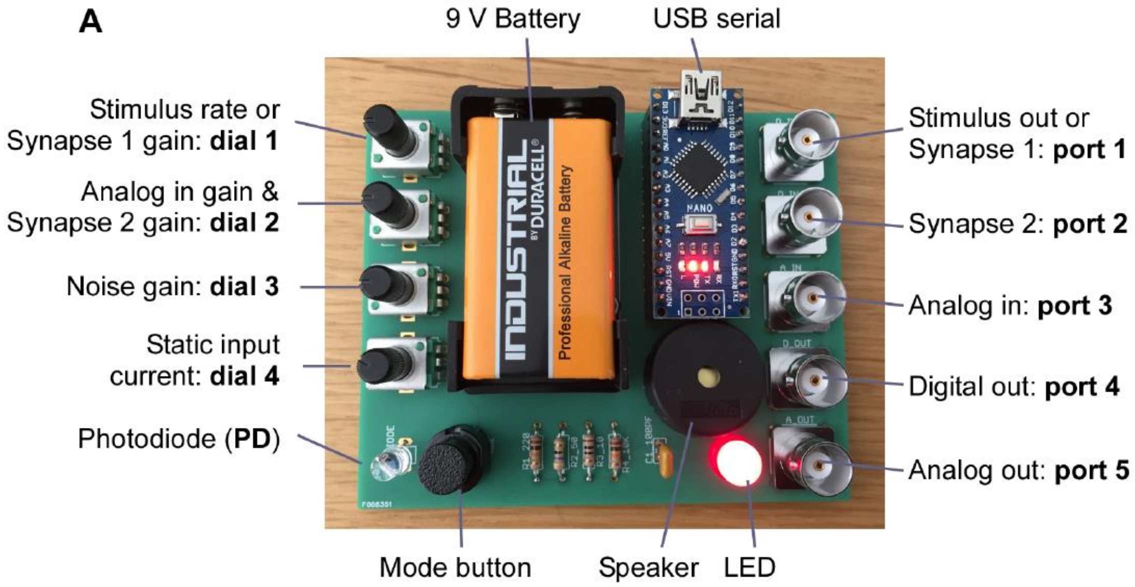


To visualise and record the activity of Spikeling, there are several options but the best is via a serial connection to a PC through the USB connection on the Arduino. Here we describe how to use the **Serial-Oscilloscope software package** provided (under creative commons from http://x-io.co.uk/serial-oscilloscope/). This lets you read all output parameters directly from the Spikeling board for display on the screen or for logging. There is also a YouTube video (https://www.youtube.com/watch?v=jgMG0UQ2_pc) demonstrating how to set up the oscilloscope.

1) Make sure the Arduino IDE is installed as you need the driver (www.Arduino.cc). Note that if you are using an Arduino-clone, the standard drivers may not work. In this case, google for the correct ones and install those instead.
2) Declare the serial port. When you plug the USB cable into the PC you may see a message telling you which "COM-port" is being used. This is the "serial port" which you need to set in the menu of the serial oscilloscope. It may just give one option which is then probably the correct one. The COM port is also found under the Device manager.
3) Set the Baud Rate to 230400.
4) The window should now produce a continuous stream of numbers. These are the output values from Spikeling. To plot them, open the oscilloscope(s) under the menu. Spikeling outputs 9 parameters in parallel, but each oscilloscope window can only display 3. If you need parameters further down the list, simply open a second oscilloscope. You can separately adjust the scale of each trace on the oscilloscope by first selecting it (click the "Beam" button) and then scaling it (left-

most thin arrows) and off-setting it (big red arrows). You can also trigger the oscilloscope using the menu on the bottom right.

5) To record data, click “log to file”. This will write a text file (comma and tab separated ASCII) with all numbers output from Spikeling. You can look at this output using any common data-analysis software (for example GNU-R, which is free, or Igor Pro or MATLAB, which are licensed).

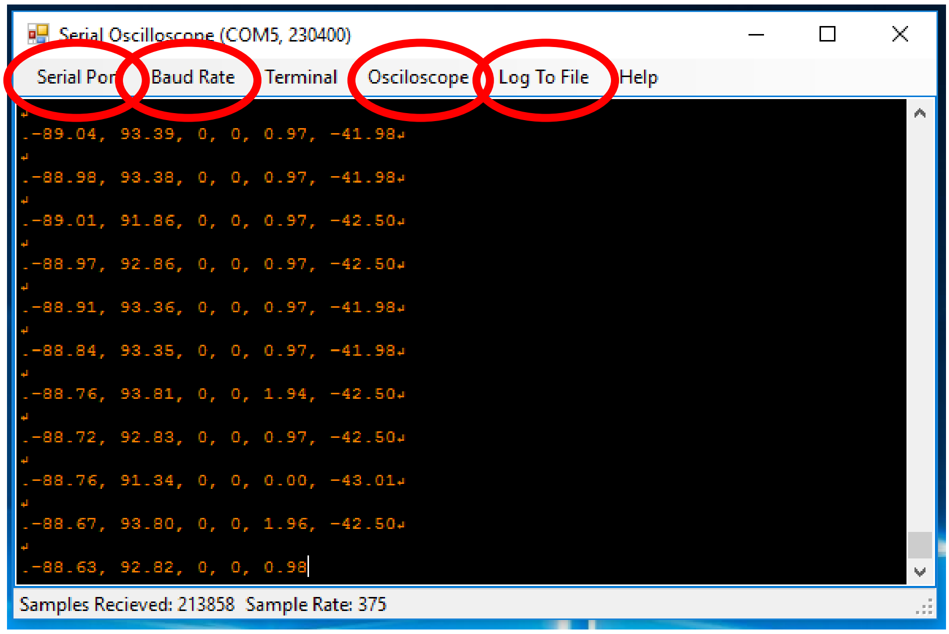


The output parameters of Spikeling are as follows:

1) Membrane voltage $V_m$ (in “mV“)
2) Total input current $I_{total}$ (in arbitrary units). $I_{total} = I_{Vm} + I_{PD} + I_{Syn1} + I_{Syn2} + I_{analogIn}$
3) Stimulus state of Synapse 1 port (see below, range 0-1)
4) Synapse 1 state (see below, boolean 0-1 for nospike / spike in the input)
5) Synapse 2 state (see below, boolean 0-1 for nospike / spike in the input)
6) Total photodiode current $I_{PD}$ (in arbitrary units)
7) Total Analog In current $I_{analogIn}$ (in arbitrary units)
8) Total Synaptic current $I_{Syn}$ (in arbitrary units) $I_{Syn} = I_{Syn1} + I_{Syn2}$
9) System time since last reset (in microseconds)

You can also read Spikeling’s Vm output via the BNC ports using a regular oscilloscope. Digital Out only carries the spikes (TTL 5V) while Analog Out on port 5 carries a version of the analog membrane voltage (in arbitrary units). (Note that this output is a pulse-width modulated (PWM) digital signal that is RC-filtered to produce and analog out: it will therefore display filtering artefacts).

***Note:*** *If Spikeling is doing “weird things” that clearly does not align with this manual, chances are there is a bad solder contact somewhere – either connecting two pins that should not be connected, or not connecting pins that should be connected (e.g. due to cold solder contacts). Check all contacts carefully and redo them if necessary (i.e. heat them up briefly so they can resettle). It can be worthwhile checking connections with a Multimeter, if available.*

Most of the time, simply displaying the 1st three outputs on oscilloscope 1 will be sufficient:

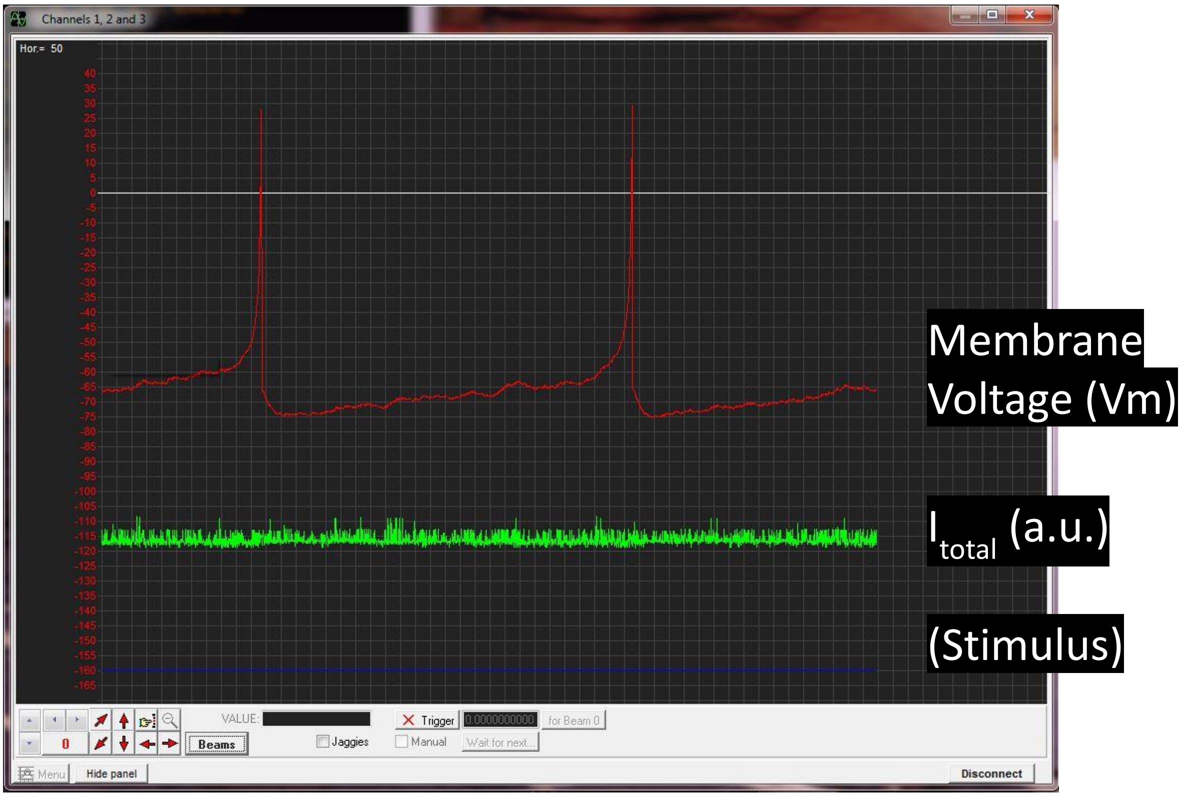


*200 ms per division*

Adjusting axes:

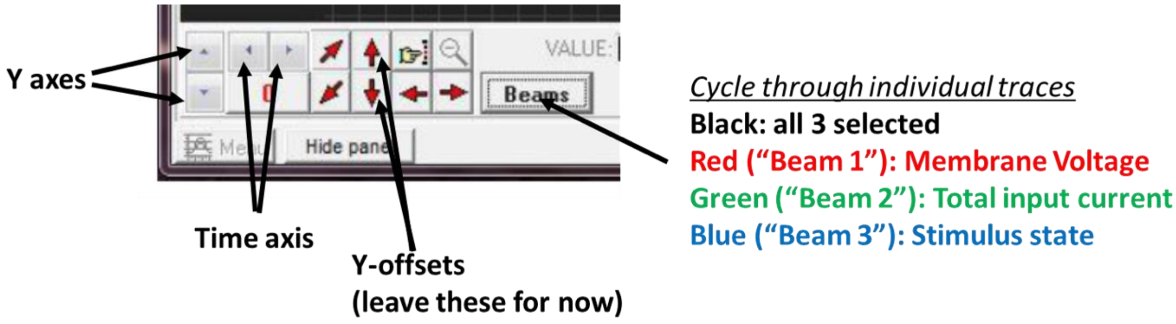


Trigger mode:

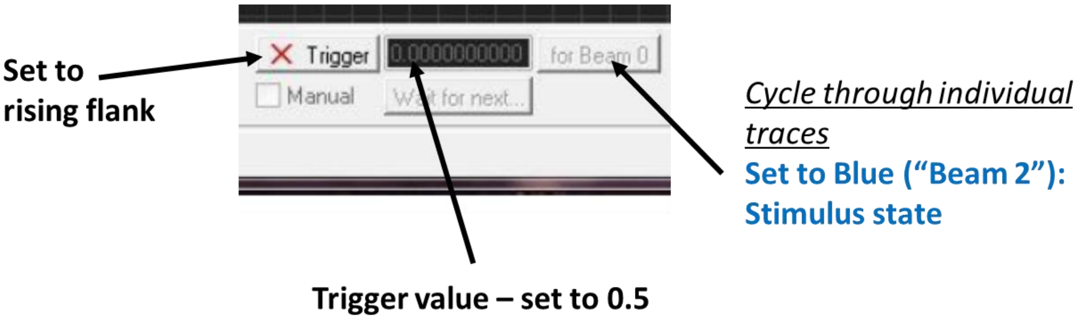

## Visualising Spikeling Output with MATLAB

The Serial Oscilloscope plotter saves recordings in comma-separated CSV files. These can be read by a wide range of software: open source possibilities include GNU-R (https://www.r-project.org), Python (https://www.python.org) or Octave (https://www.gnu.org/software/octave/). To comply with software preinstalled on the teaching PCs available at the University of Sussex, we chose to use MATLAB (Mathworks). MATLAB licenses are, however, costly and below we also provide instructions on the use of Python (free!) for visualising Spikeling output.

After opening MATLAB, make sure you have the recorded data and the MATLAB scripts in the same folder. Navigate to that folder (alternatively, the scripts/data may be added to the path.)

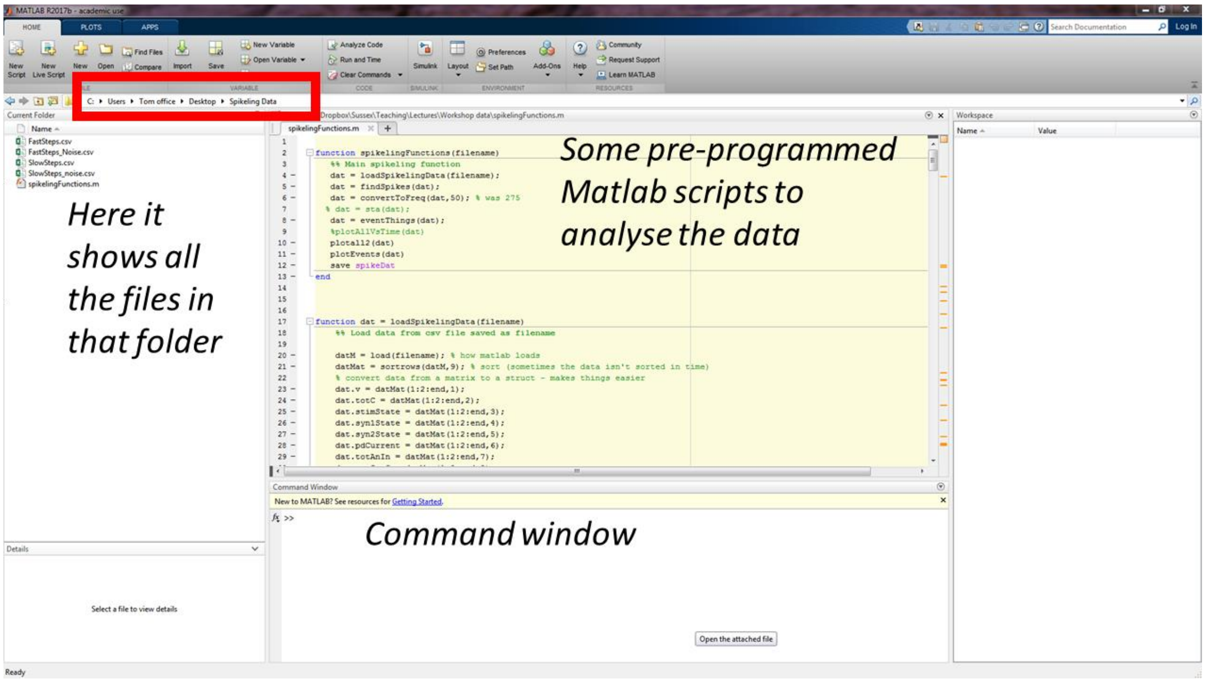


→ **In the command window, type:**

spikelingFunctions("filename.csv")

*(where filename is the name you gave your recording)*

*This will execute a series of pre-programmed plot and analysis functions that should open in separate windows. For now, lets just look at the 1st one that opens, the overview plot.*

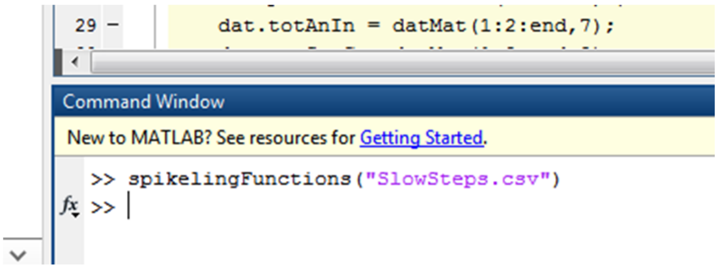

## Opening Data in Matlab:

### → Navigating the overview plot

*This plots the entire recording in your file, and first of all plots all key parameters.*

*The raw voltage trace*

*The currents, broken down into individual constituents*

*The stimulus state and any incoming spikes on Synapse 1 and 2*

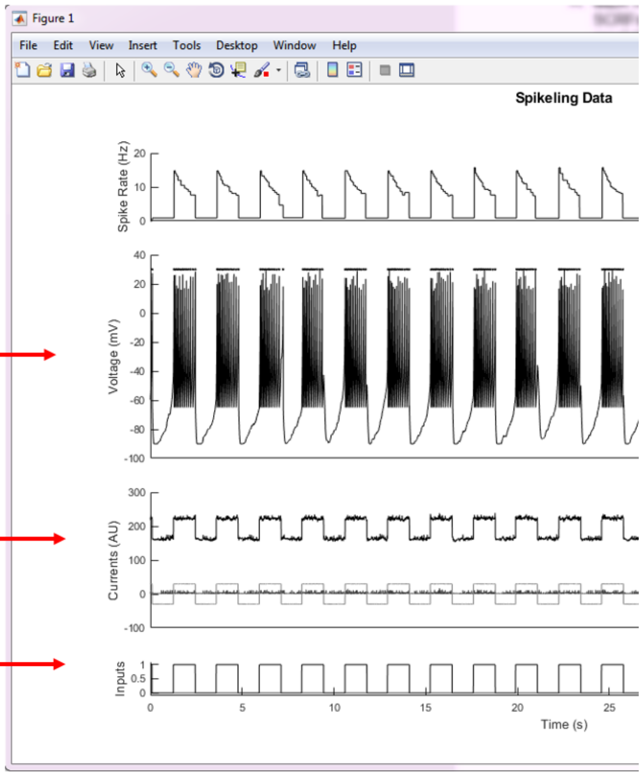


## Opening Data in Matlab:

### → Navigating the overview plot

*In addition, it automatically computes a few handy things:*

*"Instantaneous spike rate". In the example, spike rate systematically drops over the course of each stimulus as the neuron adapts.*

*On top of the raw voltage trace, it plots also each detected spike as a dot. This is useful for raster plots.*

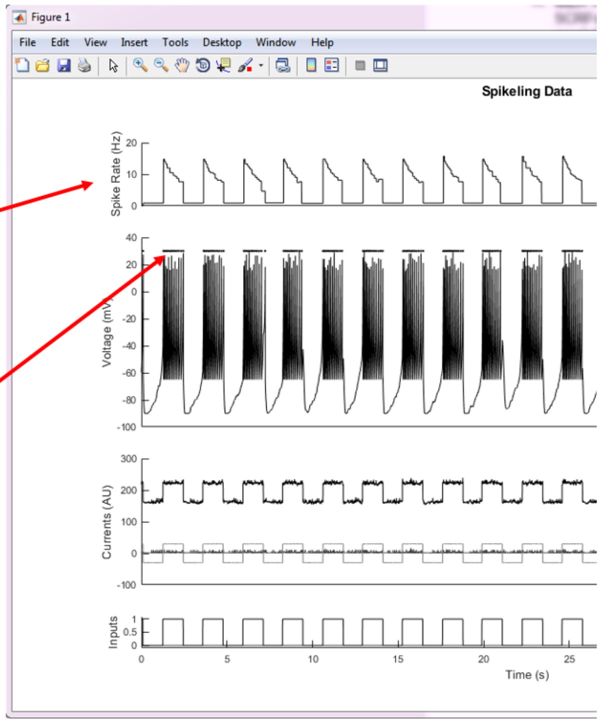

## Opening Data in Matlab:

### → Navigating the overview plot

*Some key manipulations you may want to use on your graph:*

*You can zoom in and out using the magnifying glasses. If you zoom in a bit, you can then use the hand icon to drag the traces about. Note that all time-axes are linked so they change together.*

*You can get individual values from any trace using the data cursor.*

*If you want to edit more heavily (e.g. remove a trace, change the axes etc) you can do so via the editor.*

*Save your figures using the disk icon as usual.*

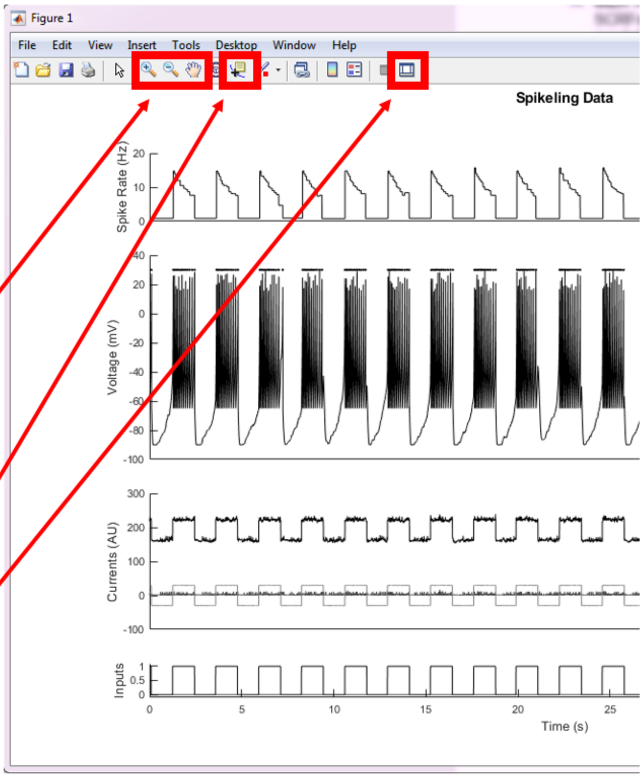


## Opening Data in Matlab:

### → Peri-stimulus analysis

*Look at the other plot that pops up. This is an automated analysis routine that finds the start of each stimulus cycle, aligns all data traces to this and computes a few more extra bits and bops.*

*Note: if you have an irregular stimulus, or no stimulus at all, this will look like chaos or be blank! In that case, just open the "SlowSteps.csv" file provided (the one used in this example).*

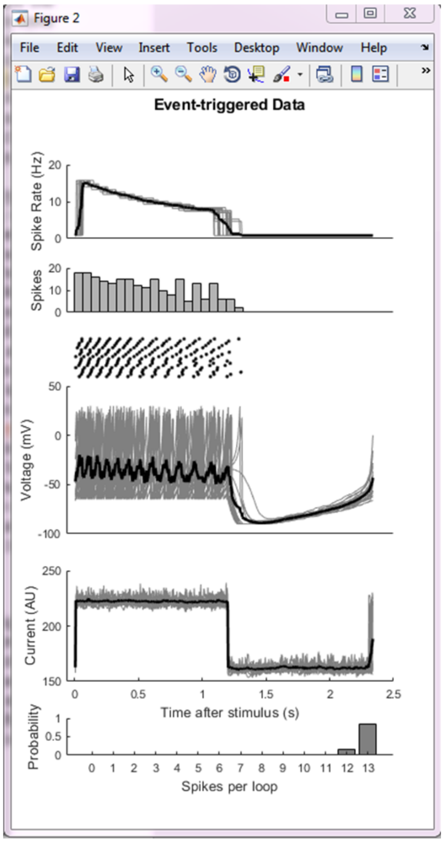

**<u>Opening Data in Matlab:</u>**

### → Peri-stimulus analysis

*Instantaneous spike rates (grey) and their mean (black)*

*A count of spikes per time-bin*

*A raster plot of spike times*

*Raw voltage traces superimposed (grey) and mean (black). Note that the mean voltage trace looks odd. This is expected, averaging raw spikes is usually a bad idea! (averaging non-spiking signals is informative, though!)*

*Total input current superimposed (grey) and mean (black)*

*It also counts the spikes per loop and plots them as a probability distribution.*

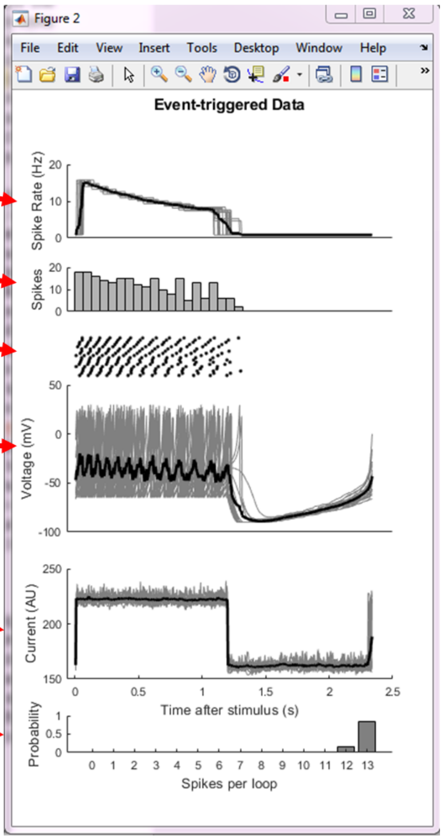


## Visualising Spikeling Output with Python

We also provide similar scripts in Python (which is free) in the form of a **Jupyter Notebook** ([http://jupyter.org)](http://jupyter.org) that reads the serial plotter's logged csv file, detects spikes, aligns the data in different ways and plots the results in simple graphs. Here we provide a brief explanation of how to run the Python code, rather than a repeat of the detailed explanation of the plot functions.

### Getting started with Python

While the Jupyter Notebook could principally also be executed directly from GitHub without installing Python, this would quickly generate issues with the way that the script provided loads the csv data. For simplicity and convenience, we therefore here give a brief account of how to execute and modify the script in **Anaconda**.

1) Download Anaconda (it's free). Navigate to [https://conda.io/docs/user-guide/install/](https://conda.io/docs/user-guide/install/), pick the right version for your operating system and follow the install instructions given
2) Once installed, launch a Jupyter Notebook, for example by executing the Anaconda Navigator and clicking "launch" under the corresponding tab. This will open a window in your web browser showing some folders on your home hard drive. Here, navigate to the folder containing the Jupyter script provided (Spikeling Analysis.ipynb) and open that script.

3) Also copy whatever csv file recorded with Spikeling you want to analyse. In the example given below, this will be the file SlowSteps1.csv in the zipped Example Data provided.
4) Your screen should now hopefully look something like this:

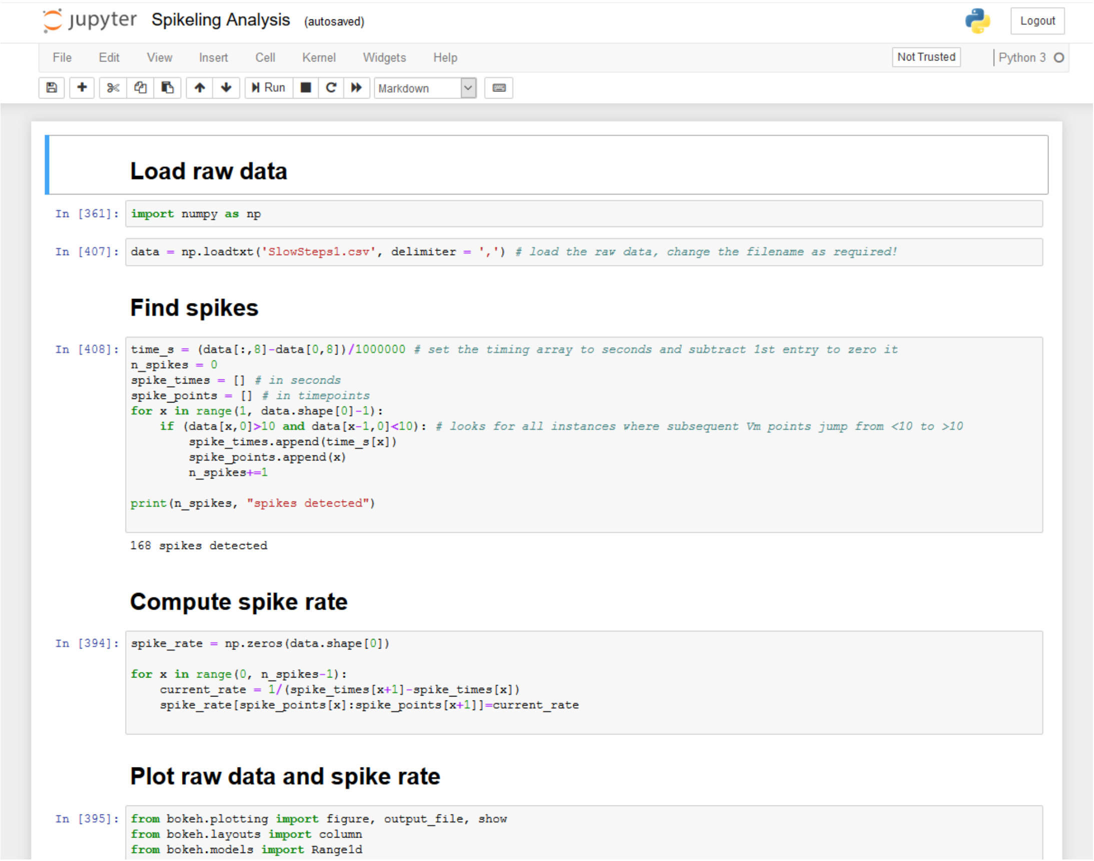


## Using the Juypter Notebook

Jupyter notebooks are organised into "cells" (each grey area) which can be called individually. Alternatively, all cells can be called at once (in sequence) to execute the whole thing.

Before doing so, make sure that the file name to be analysed is entered where it says:

data = np.loadtxt('SlowSteps1.csv', delimiter = ',')

*i.e. if you wanted to read a file name "SomeNewData27.csv" that line should read:*

*data = np.loadtxt('SomeNewData27.csv', delimiter = ',')*

Now, click on the topmost cell (i.e. where it reads "Load raw data") and then click "run". This will execute that one cell and jump to the next (which in this case it doesn't do anything interesting). If you now keep clicking "run", it will execute each cell of the script in sequence.

*To run the whole thing in sequence (not ideal as the bottom of the script has some optional bits) you can also press the >> button 3 steps to the right of "Run"*

Once you reach the first plot, it should open up a new tab that simply plots the raw data (and detected spike times). It should hopefully look something like this:

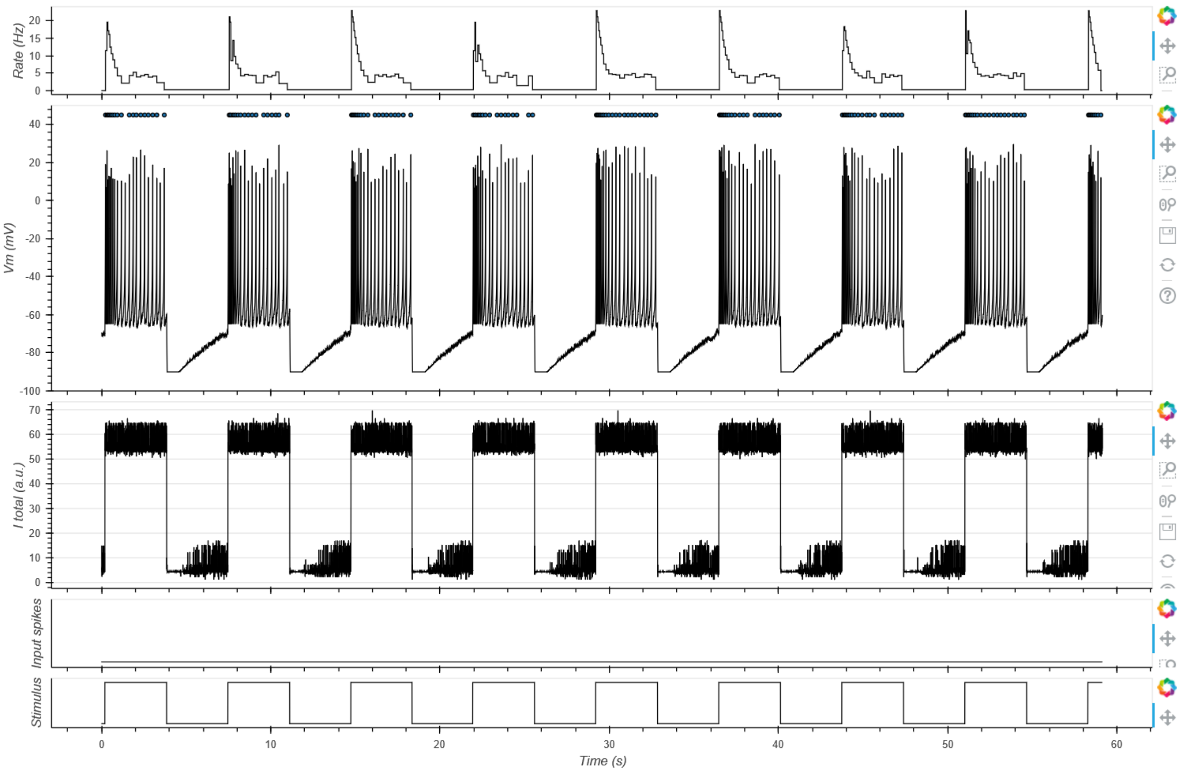


You can modify & save these plots by using the commands on the right.

Note: Depending on your screen resolution, the bottom/right of the plot may be cut off. To fix this, change the declared size of the window(s) it opens in the plot routine. In the script:

## Plot raw data and spike rate

```
from bokeh.plotting import figure, output_file, show
from bokeh.layouts import column
from bokeh.models import Range1d

output_file("RawDataPlot.html")

spike_plot = figure(plot_width=1200, plot_height = 100)
spike_plot.line(time_s[:],spike_rate[:], line_width=1, line_color="black") # Spike rate
spike_plot.yaxis[0].axis_label = 'Rate (Hz)'
spike_plot.xgrid.grid_line_color =None
spike_plot.ygrid.grid_line_color =None
spike_plot.xaxis.major_label_text_font_size = '0pt'  # turn off x-axis tick labels
spike_plot.yaxis.minor_tick_line_color = None  # turn off y-axis minor ticks

vm_plot = figure(plot_width=1200, plot_height = 300, y_range=Range1d(-100, 50),x_range=spike_plot.x_range)
vm_plot.line(time_s[:],data[:,0], line_width=1, line_color="black") # Vm
vm_plot.scatter(spike_times[:],45, line_color="black") # Rasterplot over spikes
```

… the lines:

Spike_plot = figure(plot_width=1200, plot_height = 100)

Means that this part of the figure will be 1200 pixels wide and 100 high. Simply change those numbers and re-execute the cell. You will have to do this for each subplot, i.e. also for vm_plot = … etc.

## Plotting Stimulus aligned data

Following execution of the subsequent the “Analysis Open 1…” cells, a few cells further down, under heading “plot stimulus aligned data” it will generate the stimulus aligned plot. The dimensions of this can be altered in the same way as described above, if required.

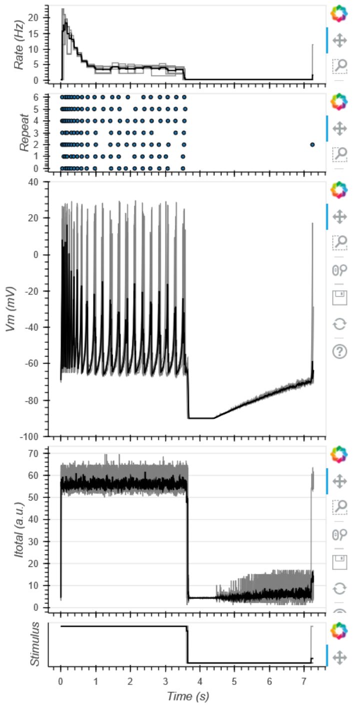


## Plotting STAs

Alternatively, if the data is a “noise sequence” generated to recover linear filters (spike triggered averages, STA), skip the whole part that says “Analysis Option 1…” and go straight to “Analysis Option 2: Spike triggered average (STA)”.. Executing this cell will give the STA:

### Analysis option 2: Spike triggered average (STA)

In [20]:

```
sta_points = 200 # number of points computed

sta_individual = []
sta_individual = np.vstack([data[x-sta_points:x,2] for x in spike_points[2:-1]])
sta = np.mean(sta_individual, axis=0)

import matplotlib.pyplot as plt
plt.plot(time_s[0:200],sta[:])
plt.ylabel('Kernel amplitude')
plt.xlabel('Time before spike (s)')
plt.show()
```

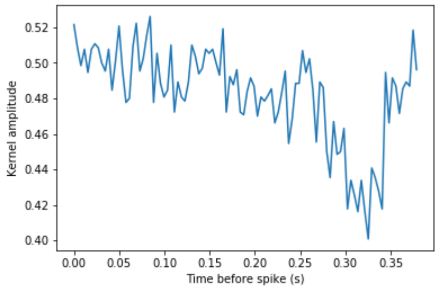


*(here shows the result for the STA of the example data “Noise_Mode3.csv”)*

## Reprogramming Spikeling (Arduino)

The "brain" of Spikeling is the Arduino-Nano microcontroller (the blue thing). By tweaking the code that is uploaded here (via the USB cable from the Arduino IDE) it is fairly straightforward to modify Spikeling behaviours, for example by modifying some of the "mode settings". When opening the Arduino code, in the top there is a list of variables and arrays with some annotations. These should be the 1st point of contact when modifying the code. After each modification, the code needs to be saved and uploaded to the Arduino for the change to take effect.

SpikelingArduinoScript

```
int   nosound        = 0;   // 0 default, click on spike + digi out port active. 1 switches both off
int   noled          = 0;   // 0 default, 1 switches the LED off
int   FastMode       = 0;   // default 0; if >0, the script is more optimised for speed by removing some of the serial outputs at the
                            // ... end of the script. This will systematically speed up the whole thing, but the system time will no longer be output as the 8th column
                            // ... meaning that the analysis scripts would need to be adjusted to reflect this (i.e. the array entry of the system time, default - column 8).
                            // FastMode = 0: Stores 8 model parameters via serial, runs at ~280 Hz, system time in column 8 (of 8)
                            // FastMode = 1: Stores 4 model parameters via serial, runs at ~390 Hz, system time in column 4 (of 4)
                            // FastMode = 2: Stores 2 model parameters via serial, runs at ~480 Hz, system time in column 2 (of 2)
                            // FastMode = 3: Stores 0 model parameters via serial, runs at ~730 Hz, DOES NOT SEND DATA TO PC!
                            // The next best thing to furhter increase speed would be to call the several analog.read/write functions
                            // less frequently. If all are disabled, the mode can exceed 1kHz, but then the dials/PD don't work... One compromise
                            // around this would be to call them less frequently. This would give a little extra speed but eventually make the
                            // dials and photodiode feel "sluggish". The latter is currently not implemented
int   AnalogInActive  = 1;  // default = 1, PORT 3 setting: Is Analog In port in use? Note that this shares the dial with the Syn2 (PORT 2) dial
int   Syn1Mode        = 1;  // default 1
                            // Syn1Mode = 0: Synapse 1 Port works like Synapse 2, to receive digital pulses as inputs
                            // Syn1Mode = 1: Synapse 1 Port acts as a Stimulus generator, with pulse frequency being controlled by Syn1Dial
                            // Syn1Mode = 2: Synapse 1 Port acts as a Stimulus generator, generating random Noise sequences (for reverse correlation)
                            // Note: this is being read into the Array_DigiOutMode Array below. This can also be manually set for each Mode, if desired by
                            // simply replacing the Syn1Mode entries in this array with 0, 1 or 2

float PD_Scaling      = 0.5;  // the lower the more sensitive.                      Default = 0.5
int   SynapseScaling  = 50;   // The lower, the stronger the synapse.               Default = 50
int   VmPotiScaling   = 2;    // the lower, the stronger the impact of the Vm poti.  Default = 2
int   AnalogInScaling = 2500; // the lower, the stronger the impact of Analog Input. Default = 2500
int   NoiseScaling    = 10;   // the lower, the higher the default noise level.      Default = 10

float Synapse_decay = 0.995; // speed of synaptic decay.The difference to 1 matters - the smaller the difference, the slower the decay. Default  = 0.995
float PD_gain_min   = 0.0;   // the photodiode gain cannot decay below this value
float timestep_ms   = 0.1;  // default 0.1. This is the "intended" refresh rate of the model.
                            // Note that it does not actually run this fast as the Arduino cannot execute the...
                            // ...full script at this rate.  Instead, it will run at 333-900 Hz, depending on settings (see top)

// set up Neuron behaviour array parameters
int   nModes = 5; // set this to number of entries in each array. Entries 1 define Mode 1, etc..

  // Izhikevich model parameters - for some pre-tested behaviours from the original paper, see bottom of the script
float Array_a[]           =  { 0.02,  0.02, 0.02, 0.02, 0.02 }; // time scale of recovery variable u. Smaller a gives slower recovery
float Array_b[]           =  { 0.20,  0.20, 0.25, 0.20, -0.1 }; // recovery variable associated with u. greater b coules it more strongly (basically sensitivity)
int   Array_c[]           =  {  -65,   -50,  -55,  -55,  -55 }; // after spike reset value
float Array_d[]           =  {  6.0,   2.0, 0.05,  4.0,  6.0 }; // after spike reset of recovery variable

float Array_PD_decay[]    =  { 0.00005,  0.001, 0.00005,  0.001, 0.00005  }; // slow/fast adapting Photodiode - small numbers make diode slow to decay
float Array_PD_recovery[] =  {   0.001,   0.01,   0.001,   0.01,   0.001  }; // slow/fast adapting Photodiode - small numbers make diode recover slowly
int   Array_DigiOutMode[] =  {Syn1Mode,Syn1Mode,Syn1Mode,Syn1Mode,Syn1Mode}; // PORT 1 setting. 0: Synapse 1 In, 1: Stimulus out, 2: 50 Hz binary noise out (for reverse correlation)
int   Array_PD_polarity[] =  {       1,     -1,      -1,      1,       1  }; // 1 or -1. flips photodiode polarity, i.e. 1: ON cell, 2: OFF cell
```

The screenshot above shows the main variables to tune if desired. For example, replacing the nosound variable currently set to "0" with a "1" will stop the clicking whenever it spikes (note it will also disable the Digi out port as these are on the same connector!).

To modify an existing "mode", you need to edit the corresponding entries in the 8 arrays listed below "Izhikevich model parameters". For example, switching the first entry in Array_PD_polarity from currently "1" to "-1" will invert mode 1's photo-response to make it an "Off-cell". In the same way, changing the "1" to a "2" will double the gain of the photo-response, etc.

To add new modes, or delete existing ones, simply set the nModes parameter to the new number of entries in each array (must be the same!) and modify the arrays accordingly. For a handy lookup of useful Izhikevich model parameters, scroll to the bottom of the code.

**Note**: Due to space constraints on the PCB, two Spikeling ports/dials are double booked by two functions. These are:

**i)** Synapse 1 & Stimulus out use the same port (1), and cannot operate at the same time. This is defined in the Array_DigiOutMode array. "0" enables Synapse 1, "1" enables the 50% duty cycle pulses digi out (default) and "2" enables the 50 Hz noise stimulus used for estimating linear filters (cf. Fig. 6).

ii) Analog In and Synapse 2 gain use the same dial (2). Usually this does not create a conflict as in most cases either Analog In and synapse 2 are not used at the same time. If a conflict arises, it is possible to switch off the Analog In gain using the AnalogInActive variable (set to 0). Advanced users may also choose to re-port these functions in the core code – this would involve resetting the port variables in "void setup" and checking that any use of these ports in the remainder of the code aligns with that change.

## Arduino Implementation of Izhikevich Model

The Izhikevich model is implemented by iteratively computing voltage (v) based on two equations:

(1) v = v + timestep_ms * (0.04 * v * v + 5*v + 140 – u + I_total);
(2) u = u + timestep_ms * (Array_a[NeuronBehaviour] * (Array_b[NeuronBehaviour]*5 – u));

and two conditionals:

(3) if (v>=30.0) {v=Array_c[NeuronBehaviour]; u+=Array_d[NeuronBehaviour];}
(4) if (v>=-90) {v=-90.0);}

```
// compute Izhikevich model
float I_total = I_PD*Array_PD_polarity[NeuronBehaviour] + I_Vm + I_Synapse + I_AnalogIn + I_Noise; // Add up all current sources
v = v + timestep_ms*(0.04 * v * v + 5*v + 140 - u + I_total);
u = u + timestep_ms*(Array_a[NeuronBehaviour] * ( Array_b[NeuronBehaviour]*v - u));
if (v>=30.0){v=Array_c[NeuronBehaviour]; u+=Array_d[NeuronBehaviour];}
if (v<=-90) {v=-90.0;} // prevent from analog out (below) going into overdrive - but also means that it will flatline at -90. Change
```

In detail:

Lines (1) and (2) are the main model implementations, where…

…we first compute the new value for voltage (v) by adding a quadratic formula of v with recovery variable (u) subtracted, followed by the addition of a single number I_total that reflects the summed input currents (from static input current, photodiode, analog in, synaptic currents etc.)

… and then we re-compute a new value of the recovery variable (u) based on Array entries Array_a and Array_b at position NeuronBehaviour (=the mode, switchable by the on-board button). At default, NeuronBehaviour == 0, so the 1$^{st}$ entries in Array_a and Array_b are used, and when pressing the button NeuronBehaviour is incremented by +1 each time until it resets if it exceeds nModes (further up in the script). The Arrays are found near the top of the script, and reflect the original parameters a, b (& c, d, see below) from the model. Generally, a larger value of u hyperpolarises the model in the next computation of u, as it directly opposes I_total.

In both lines (1) and (2), the increment to v and u is scaled by global variable timestep_ms (default = 0.1). This reflects the "*intended*" model refresh rate. So, if that set of equations were called 10 times per millisecond (i.e. at 10 kHz) the model would run at "real-time". However, the actual call-rate is much lower, as the Arduino cannot execute the full script at 10 kHz. In standard configuration, it actually runs at only ~420 Hz. Ways to increase speed are discussed below ("Spikeling Refresh Rate").

Next, lines (3) and (4) are two conditionals that are triggered as a spike occurs (3) or if membrane voltage drops too low (4).

Line (3) is a carbon copy version of the original Izhikevich model, where upon execution of a spike (v exceeding 30 mV) the model resets the new value of v to be equal to Array_c[NeuronBehaviour] and resets the value of u according to Array_d[NeuronBehaviour].

Line (4) is a custom addition to the model that stops it from hyperpolarising below -90 mV. This is non-essential, but stops the model from crashing if, for instance,I_total becomes too negative to drive unrealistically low membrane voltages.

The functions of Array entries a, b, c and d are explained in detail in the original paper (Izhikevich 2003). The paper and later online additions also give a list of possible “useful combinations” of a, b, c and d that together give the model different “realistic” behaviours. For convenience they are also pasted into the bottom of the Arduino script. To implement them, simply take a specific behaviour’s 1st 4 numbers and add them to the four arrays in the top of the script:

```
// set up Neuron behaviour array parameters
int   nModes = 5; // set this to number of entries in each array. Entries 1 define Mode 1, etc..

  // Izhikevich model parameters - for some pre-tested behaviours from the original paper, see bottom of the script
float Array_a[]          =  { 0.02,  0.02, 0.02, 0.02, 0.02 }; // time scale of recovery variable u. Smaller a gives slower recovery
float Array_b[]          =  { 0.20,  0.20, 0.25, 0.20, -0.1 }; // recovery variable associated with u. greater b coules it more strongly
int   Array_c[]          =  {  -65,   -50,  -55,  -55,  -55 }; // after spike reset value
float Array_d[]          =  {  6.0,   2.0, 0.05,  4.0,  6.0 }; // after spike reset of recovery variable
```

```
// From Iziekevich.org - see also https://www.izhikevich.org/publications/figure1.pdf:
//      0.02      0.2     -65      6       14 ;...     % tonic spiking
//      0.02      0.25    -65      6       0.5 ;...    % phasic spiking
//      0.02      0.2     -50      2       15 ;...     % tonic bursting
//      0.02      0.25    -55     0.05     0.6 ;...    % phasic bursting
//      0.02      0.2     -55     4        10 ;...     % mixed mode
//      0.01      0.2     -65     8        30 ;...     % spike frequency adaptation
//      0.02      -0.1    -55     6        0  ;...     % Class 1
//      0.2       0.26    -65     0        0  ;...     % Class 2
//      0.02      0.2     -65     6        7  ;...     % spike latency
//      0.05      0.26    -60     0        0  ;...     % subthreshold oscillations
//      0.1       0.26    -60     -1       0  ;...     % resonator
//      0.02      -0.1    -55     6        0  ;...     % integrator
//      0.03      0.25    -60     4        0;...       % rebound spike
//      0.03      0.25    -52     0        0;...       % rebound burst
//      0.03      0.25    -60     4        0  ;...     % threshold variability
//      1         1.5     -60     0      -65  ;...     % bistability
//        1       0.2     -60     -21      0  ;...     % DAP
//      0.02      1       -55     4        0  ;...     % accomodation
//     -0.02      -1      -60     8        80 ;...     % inhibition-induced spiking
//     -0.026     -1      -45     0        80];        % inhibition-induced bursting
```

## Photodiode Adaptation Speed

The photodiode is inherently noisy, so here we use a “buffered time-averaging” of its signal to ameliorate some of the noise. Instead of just reading it’s state on the corresponding AnalogIn Pin (1st line, PDVal) and sending that drive directly into the model as I_PD, we instead send each instance of PDVal into a buffer array PDVal_Array of size 10. Each time the buffer is full (after 10 instances of PDVal being stored in the buffer), we take its mean (PDVal_smoothed) and use that number to inform I_PD.

Next, the Photodiode also has two gain factors (global scaling, and local gain) and two decay constants (time dependent) associated, which allows to have the PD current adapt over time, as it would in most sensory neurons.

- PD_Scaling is global and set at the beginning of the script. This is a single number that scales the amplitude of all PD_related amplitude processes
- PD_gain is the current gain of the PD system. At default, this is 1, but it falls below 1 if the PD in continuously driven, and recovers thereafter as a function of the time-dependent variables, both of which are stored in Arrays at the top of the script and depend on the Mode variable NeuronBehaviour:
    - Array_PD_decay[NeuronBehaviour]
    - Array_PD_recovery[NeuronBehaviour]
- PD_gain is limited at the top at 1 (full gain), and and the bottom at PD_min (default = 0, which will also have I_PD at 0).
- Finally, as I_PD is being used in the Izhikevich model (see previous section) it is multiplied by either 1 (no change) or -1 (invert), to allow flipping its polarity as a whole thus mimicking “On” and “Off” type behaviour, respectively. This flip is controlled by a third array: Array_PD_Polarity[NeuronBehaviour]

```
// read Photodiode
int PDVal = analogRead(PhotoDiodePin); // 0:1023
if (PD_integration_counter<10) {   // PD integration over 5 points
  PD_integration_counter+=1;
} else {
  PD_integration_counter=0;
}
PDVal_Array[PD_integration_counter]=PDVal;
PDVal_smoothed=(PDVal_Array[0]+PDVal_Array[1]+PDVal_Array[2]+PDVal_Array[3]+PDVal_Array[4]+PDVa
I_PD = ((PDVal_smoothed) / PD_Scaling) * PD_gain; // input current

if (PD_gain>PD_gain_min){
  PD_gain-=Array_PD_decay[NeuronBehaviour]*I_PD; // adapts proportional to I_PD
   if (PD_gain<PD_gain_min){
    PD_gain=PD_gain_min;
  }
}
if (PD_gain<1.0) {
  PD_gain+=Array_PD_recovery[NeuronBehaviour]; // recovers by constant % per iteration
}
```

Photodiode-related arrays at the top of the script:

```
float Array_PD_decay[]    = { 0.00005,  0.001, 0.00005,  0.001, 0.00005  }; // slow/fast adapting Photodiode - small numbers make diode slow to decay
float Array_PD_recovery[] = {   0.001,   0.01,   0.001,   0.01,   0.001  }; // slow/fast adapting Photodiode - small numbers make diode recover slowly
int   Array_PD_polarity[] = {       1,     -1,      -1,      1,       1  }; // 1 or -1, flips photodiode polarity, i.e. 1: ON cell, 2: OFF cell
```

## Model Noise

Spikeling allows adding synthetic noise to the model, to mimic noisiness in neurons. The noise is added to the I_total variable read in the Izhikevich model as I_Noise. The dial (3) controls the amplitude of the noise around 0 (NoiseAmpl), but has no impact on it's temporal statistics. This noise function was added in a fairly simple way: At each model instance a single variable I_Noise is increased/decreased by a random amount as a function of NoiseAmpl, followed by a "dampening" step of multiplying the result by 0.9. The dampening step is used to ensure the Noise parameter slowly tends towards 0, as otherwise random noise fluctuations would accumulate to drive sustained de- or hyperpolarising current. Accordingly, the temporal statistics of the noise depend on model refresh rate (see corresponding paragraph below) and this arbitrary dampening factor. We found that a value of 0.9 produces "realistic-looking" noise.

```
NoisePotVal = analogRead(NoisePotPin); // 0:1023, Vm
NoiseAmpl = -1 * ((NoisePotVal-512) / NoiseScaling);
if (NoiseAmpl<0) {NoiseAmpl = 0;}
 I_Noise+=random(-NoiseAmpl/2,NoiseAmpl/2);
I_Noise*=0.9;
```

## Spikeling Refresh Rate

The Izhikevich model works by iteratively computing v and u as a function of each other and model parameters a, b, c and d (see corresponding paragraph above). Each time this set of equations is called, the model advances by one unit of time. Accordingly, the more frequently the main loop of Spikeling can execute ("void loop"), the faster the model can run. However, this main loop includes several pieces of code that execute "slowly" (100s of microseconds), limiting the refresh rate. In "standard configuration" (i.e. all parameters set as described in the paper and as provided in the Arduino sketch), the model executes at 420 Hz as 1 full execution round of the void loop takes ~2.4 ms. This leads to a potential conflict: if the Izhikevich time-step parameter (timestep_ms, see above) were set to 2.4 ms to try to achieve maximum speed of the model, it would in fact fail because the model is set-up to mimic fast action potential generation in the mammalian central nervous system where a single spike typically lasts 1 ms at most.

Instead, to get realistic (if slowed down) waveforms of action potentials and subthreshold events, the model should be executed at an intended model rate of ~10 KHz (i.e timestep_ms = 0.1, the default value). Although the model will be executed in 0.1 ms instances, these instances are only called every 2.4 ms so that the model runs ~24 times slower than intended. The waveforms of spikes and subthreshold events continue to look realistic to the observer, they are simply a bit slow. One way to think of this might be to consider Spikeling output as mimicking neurons of cold-blooded animals, where these types of kinetics are commonplace.

## Speeding up the model

If desired, the user can speed up the execution of the model in several ways. Simply put, there are 2 main factors slowing down the loops: Serial Print operations and Analog Read/Write operations. Accordingly, reducing the number of these operations, or the frequency at which they are called will speed up the model.

***Serial Print*** operations are used at the end of the script to log model data via the USB connection to the computer. At default, it logs 9 parameters, in this order:

- Voltage v
- Total current I_Total

- Model Stimulus State Stim_State
- Synapse 1 State (spikes in?) SpikeIn1State
- Synapse 2 State (spikes in?) SpikeIn2State
- Photodiode current I_PD
- Analog input current I_AnalogIn
- Total synaptic current (1+2) I_Synapse
- System time in microseconds currentMicros

Removing some of these from the data logging can increase model speed. If all are removed (no communication with computer), the model refresh rate more than doubles to ~1,000 Hz (loop time ~1 ms).For convenience, we pre-configured a simple option to remove them in several instances through the global variable FastMode near the top of the script. At default (=0), all 9 parameters are logged, and the model runs at ~420 Hz. If set to 1, 2, or 3 the model starts dropping the later parameters, as explained in the script itself (1.57, 1.22, 1.0ms, respectively, corresponding to 633, 819 and 1000 Hz)

*(Note: The analysis scripts (Python/Matlab) provided assume that system time, which is always needed for time-accurate plotting, is stored in the 9th column (i.e. 8, with 0 indexing). However, in FastMode=1 or =2, this jumps to column 4 and 2, respectively, so if these modes are used for logging the pre-processing scripts should be updated to reflect this.)*

***Analog Read/Write*** operations are called several times within the main loop, either reading the state of a dial, the photodiode or the analog in port (3). Analog write operations drive the Analog out port (5) and the onboard LED. The user could choose to simply disable one or more of these functions. A short explanation how one might go about this is included in the sketch itself. Advanced users may also consider not disabling them, but rather calling them less frequently (e.g. only every $10^{th}$ time the model loops). While this should principally work, note that it would selectively slow down the instances in which the call was executed, so the model would not run at a constant speed but instead jump a bit. However, this may not be a big problem depending on the intended use. Notably, in the Spikeling 2.0 version (see below) we already down-scaled the Analog read bit depth to 16 to speed them up a little (Using AnalogReadHelper)

***Timestep_ms***. Finally, the user can also alter the timestep_ms global variable near the top of the script. Increasing this will speed up the Izhikevich model in a linear dependence (i.e. doubling timestep_ms will double model speed) but run risk that it skips spikes and generate weird-looking waveforms. Vice versa, decreasing it will generate “nicer” waveforms, but at further cost to speed. Notably, this will only affect the actual spike model, not the whole script.

## Spikeling 2.0

We are currently working on a new version of Spikeling (2.0) that will feature a range of improvements, including perhaps most notably the possibility to connect a TFT colour display for live display of the model output.

The new version replaces the low-cost Arduino Nano microcontroller of Spikeling 1.0 with the more modern ESP8266 (e.g. https://www.adafruit.com/product/2471). First tests indicate that the ESP8266 (by itself, without TFT screen connected) executes the Izhikevich model at about 5-10 times the speed of Spikeling 1.0 (3.7 kHz at full output, compared to 420 Hz for Spikeling 1). This can be pushed to near “realtime” with fastmode enabled (see above), such that each

loop execution round approaches 0.1 ms, which is the iteration step that the Izhikevich model is set to. Of course, it is possible (up to a point) to lower that timestep_ms parameter to match the the actual execution step to achieve “realtime”.

With the screen connected, Spikeling 2.0 speed drops to about the same speed as Spikeling 1.0.

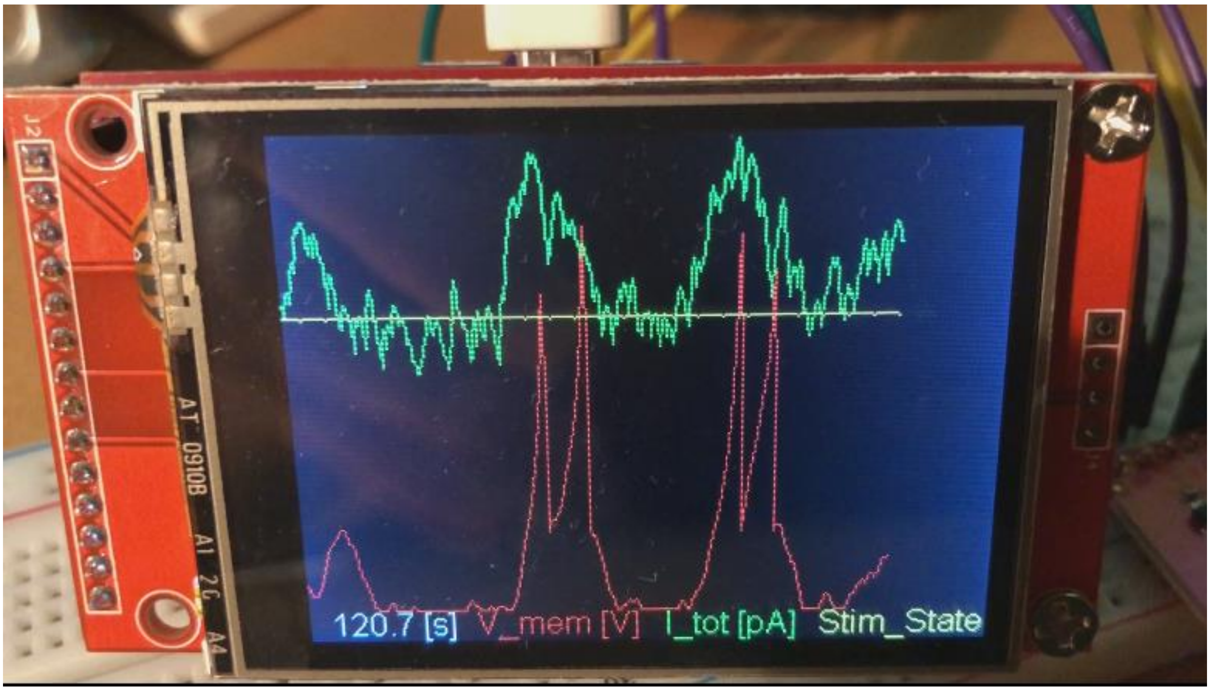


*Spikeling 2.0 prototype with TFT screen connected*

In addition to either offering speed enhancements or the option for a TFT screen, the new microcontroller is also WiFi enabled which presents the possibility to do remote-data logging without need for a dedicated USB cable. Notably, to connect the ESP8266 to the dials and photodiode used in Spikeling 1.0 requires addition of a dedicated integrated circuit (IC) chip such as the MCP3008 (e.g. https://www.adafruit.com/product/856).

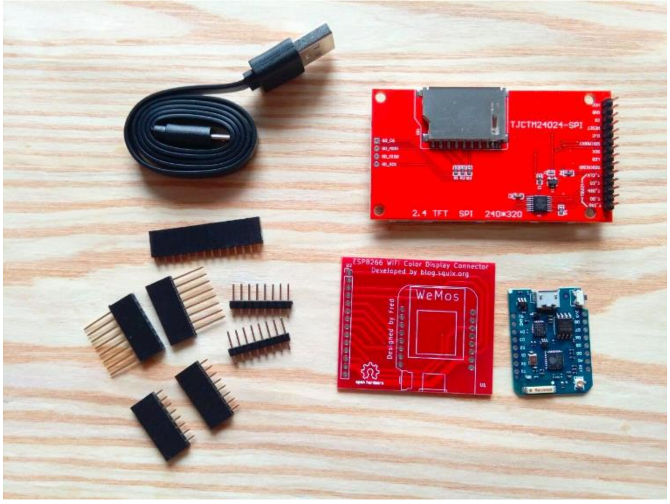

*The ESP8266 Wifi Colour Display Kit sold by ThingPulse, which includes the ESP8266 itself, a breakout board and the TFT screen:*

*https://thingpulse.com/product/esp8266-wifi-color-display-kit-2-4/*

At time of writing, Spikeling 2.0 is under ongoing development. All documentation to its current state can be found on the Spikeling GitHub: https://github.com/BadenLab/Spikeling. However, the Arduino code running Spikeling

1.0 has already been set-up to work with either version, with one required user alteration depending on which board is used:

//#include "SettingsArduino.h" ← decommented, so disabled

#include "SettingsESP.h" ← currently Spikeling 2.0 is enabled.

To switch, just move the // (decomment) to the other line

# Spikeling Exercises

## Resting membrane potential

In the absence of a stimulus, the Spikeling rests at -70 mV and should only spike sporadically. Resting membrane voltage (Vm) can be set indirectly with bottom-most dial (dial 4), which sets a constant input current. For now, on the oscilloscope we are only interested in the membrane potential trace (the red one) and the current trace (the green one). The red LED on the board also tracks Vm, and flashes with each spike which should also be accompanied by a "click". Electrophysiologists often connect a speaker to their recording of membrane voltage to get a direct audio feedback of what the neuron might respond to.

***Task 1: What happens when you increase or decrease the static input current?***

*You should observe that increasing the static input current drives Vm towards and beyond spike threshold. As you keep driving Vm upwards, you will elicit progressively higher spike rates. This is the simplest of all* ***neuronal codes*** *- the intensity of a stimulus (here, simply the increased input current) is* ***encoded*** *in the frequency of spikes. Imagine you are the postsynaptic neuron and all you see is this spike pattern – you could easily infer from seeing more spikes in close succession that the input to the presynaptic neuron has probably increased. Most spiking neurons use this* ***rate code*** *to signal input intensity.*

*On the screen, note that each spike is preceded by a shallow rise in Vm and followed by a brief dip below starting levels. This dip is the refractory period of the neuron. During this time, generating another spike is particularly difficult. At the extreme low point of Vm it is impossible to generate a spike, which in a biological neurons is because the sodium channels are blocked (not just closed). This* ***absolute refractory time,*** *together with the duration of the spike itself (1-2 ms) sets a limit on the maximum spike rate possible. In an average neuron, the absolute refractory period is a few milliseconds, and thus the maximal spike rate of most neurons is ~100-200 Hz. Some specialised neurons can go a bit higher, but kHz range is out of question. This means that, by using a single spiking neuron, it is impossible to faithfully encode a time-varying stimulus above this frequency. However, there are a few tricks around this problem that the nervous system can use. We will pick up on this point later.*

***Task 2: What happens when you dial current up and then wait a few seconds?***

*If you drive up input current and leave it there for a few seconds, you should observe that spike rate first increases, but then will taper off to some new basal rate of activity which will be higher than the original rate (rate code), but lower than the peak rate. This is an example of* ***adaptation****. Neurons respond to a change in the input not only by firing more or fewer spikes, but in addition by adjusting their sensitivity to further changes based on recent stimulus history. This is a fundamental property of neurons that allows them to extend their operating range, and to stay responsive to further changes in subsequent inputs.*

***Task 3: Does a rapid and a slow current increase generate the same voltage response?***

*As you increase input current slowly or rapidly, you should observe that you can reach different peak spike rates. A rapid increase in input current is a much more effective way to trigger multiple spikes in close succession. This is again because of adaptation. If you change input current fast enough, the neuron does not have time to adapt and therefore fires vigorously at first. If you change input current slowly enough, you should be able to drive it quite high without eliciting many extra spikes as it adapts while you slowly ramp up the current. This means that not only the absolute level of a stimulus can be encoded by a neuron, by also the rate of change. Note that this creates ambiguity in the code, which is one important reason for the need of* ***parallelisation****. This means that if you want to read both absolute levels of a stimulus and its rate of change, you may need two neurons with different properties.*

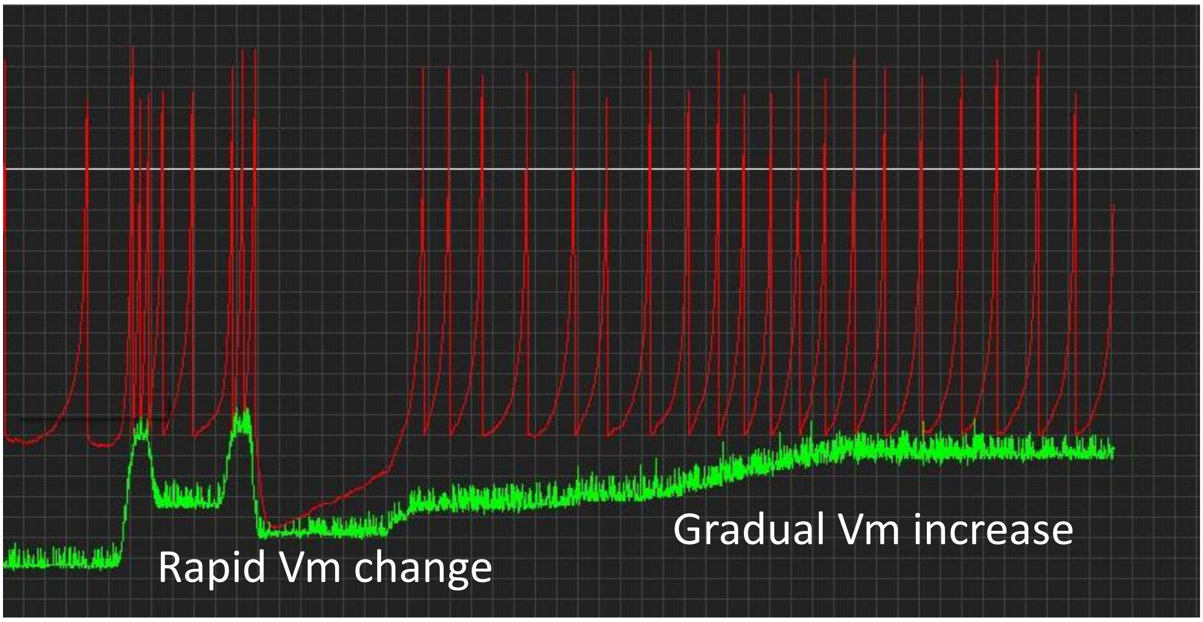


*NB: The fact that the speed of change in the input is encoded in a neuron's firing also means that that spike thresholds are not fixed. Depending how quickly you stimulate a neuron, it can start firing at different Vm values!*

## External Stimulation: Light

Spikeling has a built-in photodiode – the clear dome-shaped object in the lower left corner. This functions like a "mini solar-panel". If you shine light at it, it generates a tiny voltage, and Spikeling is programmed to react to this voltage. Increasing the amount of light hitting the photodiode drives a depolarising current, just like the static input current dial did (above).

***Task 4: Shine some light at the photodiode e.g. using a torch or by holding it to the room light. Observe both the current (green) and the voltage trace (red). Can you get the cell to spike in response to light? Does it spike every time you hold it into the light?***

*Shining light at the photodiode should cause a brief increase in the driving current, which will be mirrored by the membrane voltage. If the light intensity is high enough, you should be able*

*to generate spike(s). However, if the light is weak, or presented only for a very short amount of time, you will probably not drive a spike. This is an example of how neurons can use a spike threshold to only report the presence of a stimulus if it is of a certain amplitude and minimum duration. However, do note that even if there is no spike generated, the membrane voltage will still react to most changes in the light. This* ***"sub-threshold" activity*** *is fundamental to many neuronal computations. We will return to this point later.*

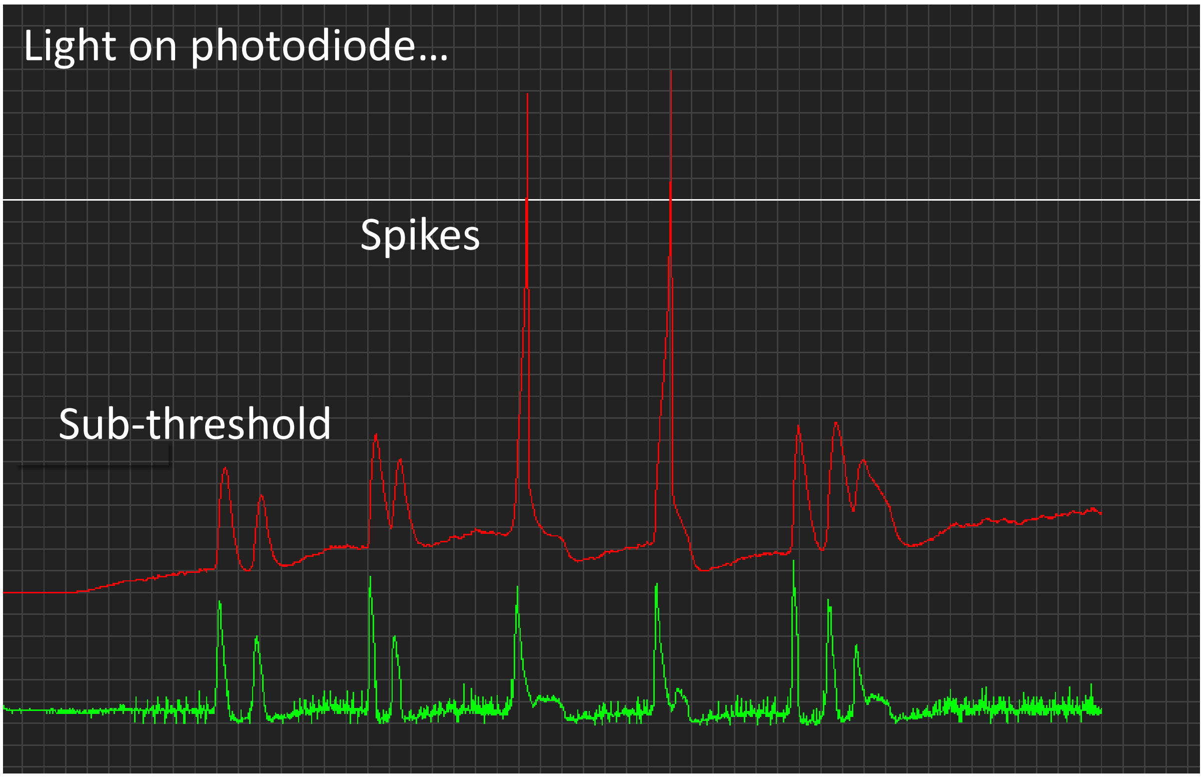


**Task 5: Turn the static input current dial up so that Spikeling generates a few spikes per second, and then shine some steady light at it. You should observe that the spike rate increases a lot at first, but then settles to an intermediate spike rate (just as before when you increased just the Vm dial). Now, suddenly remove the light. What happens?**

*You should observe that when you remove the light, membrane voltage will drop not just back to baseline levels, but below baseline for a short period of time. As it drops below baseline, it will probably result in a brief gap in spikes. This is an example of how a sudden absence in a stimulus that the system has adapted to can be encoded by the neuron by the absence of expected spikes. However,* ***the salience of this code is low****. For a postsynaptic neuron reading this signal, it is much easier to respond to the presence of an unexpected spike, rather than the absence of an expected one. (How could you turn the absence of an expected signal into the presence of an unexpected one?)*

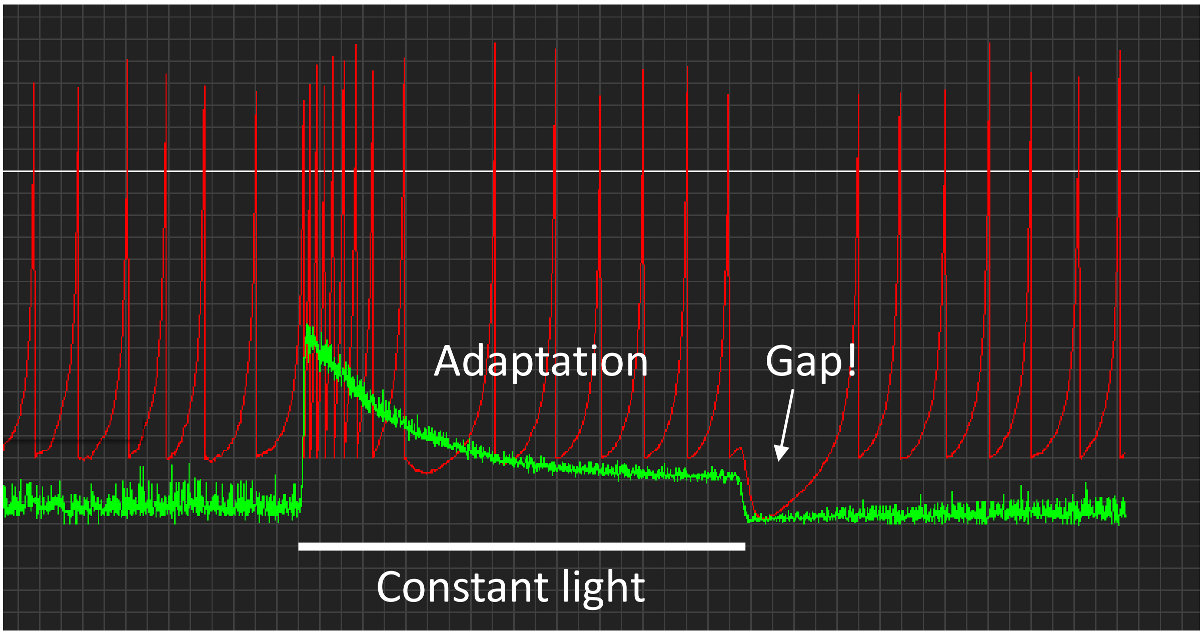


## Switching spike-modes

Spikeling comes with multiple preprogrammed behaviours, which can be cycled through with the on-board button (the big black one). If you press the button from reset state, a little LED on the Arduino should light up twice. This means that Spikeling is now in “Mode 2”. If you press it again, it should blink 3 times (Mode 3) and so on. If you get to the end (Mode 5), it will cycle back to 1. You can always jump to 1 by resetting the Arduino (the little white button on the Arduino itself).

The preprogrammed modes are as follows:

1) Regular spiking neuron, slow adapting photodiode (“Sustained ON”)
2) Bursting neuron, fast adapting inverted photodiode (“Transient Bursting OFF”)
3) Fast spiking neuron, slow adapting inverted photodiode (“Sustained OFF”)
4) “Chattering“ neuron, fast adapting photodiode (“Transient ON“)
5) High threshold firing, slow adapting photodiode (“Sustained ON II”)

For now, let’s just focus on the first 3 modes. Compare the above exercises on resting membrane voltage and photodiode stimulation in different spike modes.

**Task 6: Comparing Modes 1and 2: What are the differences when you depolarise the neurons using the static input current (dial 4) and when you pass a torch over the diode?**

*The baseline spiking behaviours, the speed at which the response to photodiode stimulation decays, and the polarity of the light response are all different. For example, if you continuously shine light at Spikeling in mode 1, it will adapt slowly. If you do the same for mode 2, it will adapt a lot faster and to a stronger degree, the current is inverted. This is an example of a* ***“transient OFF”*** *(mode 2) and a* ***“sustained ON”*** *neuron (mode 1). In the nervous system the transience of a neuron is one fundamental ingredient in generating different functions. For*

*example, transient neurons are usually good at **encoding the onset of a stimulus**, but not very useful in signalling when that stimulus stops. In contrast, the sustained neuron encodes both events quite reliably, but the **energetic cost is higher** (it needs more spikes) and the **information content per spike is lower**. Neurons are amongst the most energetically costly cells in the body. They are also amongst the most fragile if energy supply is low (for example during a stroke!). Most neurons will die after even a few seconds to minutes of oxygen deprivation. Accordingly, when considering neurons and neuronal networks, it can often be instructive to consider the energy costs associated with a particular computation. If you can implement the same computation using fewer spikes, or fewer neurons, that is probably a good thing, and most of the time it is what the brain will have evolved to do. Accordingly, it is not always about setting up the "best" computation, often it is about building the cheapest system that still works with adequate reliability.*

*Another reason for using a transient neuron is that after it signals the start of an event, it is rapidly "ready" to signal the start of another event. If a transient neuron spikes twice, it probably means there were two events. If a sustained neuron spikes twice, it could mean there are 2 events or that there was one event which is still ongoing. Accordingly, the sustained neuron's **code** is usually more **ambiguous**. Ambiguity in coding is almost always a bad thing. It reduces the information content per spike (costly!) and it usually means you need additional neurons to resolve the ambiguity (also costly).*

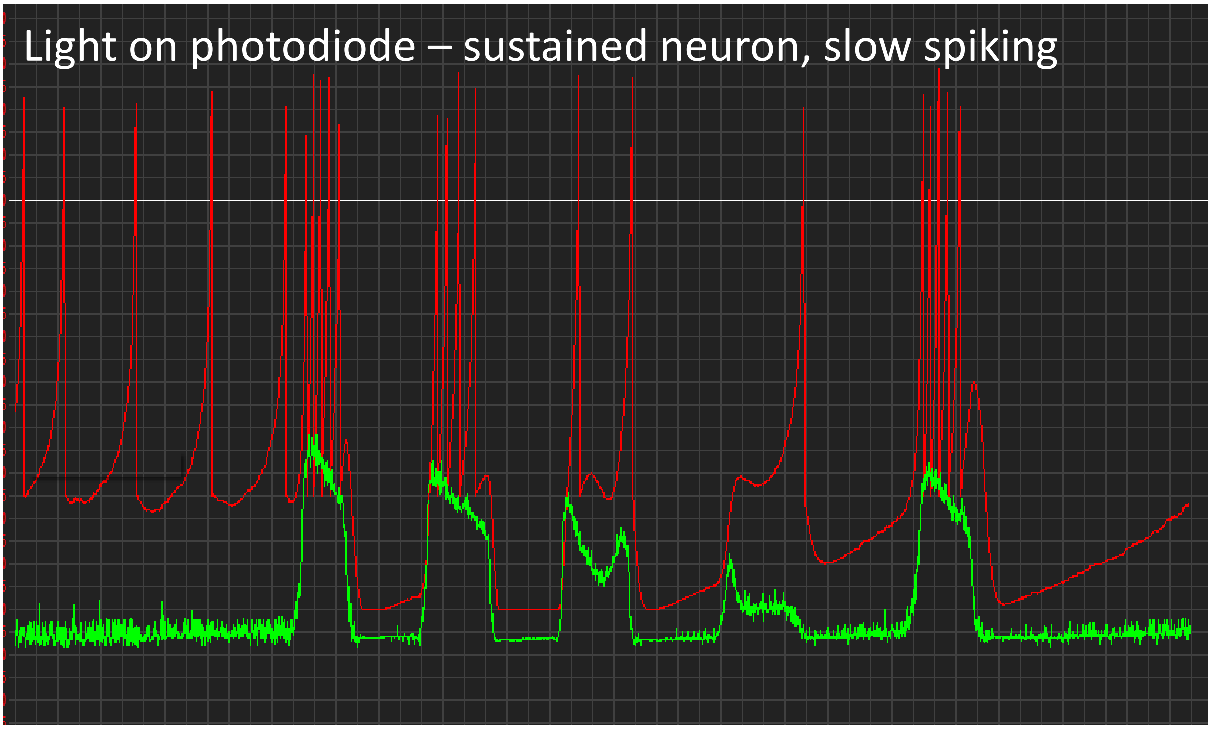

**Task 7: Compare Modes 1 and 3 at the level of the photoresponse and during current injection. What sets them apart?**

*In Mode 3, the spike threshold is lower and the refractory period following a spike is shorter. This neuron will do most things that the Mode 1 neuron will do, but it will use more spikes. In addition, its photo-response is inverted ("sustained Off cell"). While energetically costly, sometimes you need more spikes. For example, for a rate code to encode as much information as possible, the bigger the rate-range is that the neuron can cover, the more* ***finely resolved can you encode signals*** *using the frequency of spikes. One often used example here are projection neurons of auditory systems.*

*Another advantage of using a neuron that can reach high spike rates is that* ***negative coding*** *becomes more useful. If the basal firing rate of a neuron is high, a reduction in this rate (for example due to the sudden absence of a depolarising stimulus, or due to the addition of an inhibitory input) can still be readily read out by postsynaptic neurons. This is easily illustrated if you repeat an experiment from above: Set the basal spike rate to a few spikes per seconds using the static input current dial, then iteratively shine a constant light at the photodiode and then remove the light. Each time you shine light, the high "dark" spike rate should drop to a new, lower spike rate, and this change should be much more obvious (and inverted) if you use the fast spiking neuron from Mode 3 compared to the regular spiking Mode 1 neuron.*

## The "Self-Stimulator"

From the exercises with the torch, you will have noticed that it is difficult to reproduce the same stimulus from trial to trial. To help you with this, Spikeling comes with an option to "stimulate itself" by generating a defined light output. Port 1, by default, is defined as an output port which delivers 5V pulses at different intervals depending on the mode. Connect a BNC cable to this port, and on the other end connect the extra BNC-cap with an LED attached. You should now see that the LED lights up in regular intervals. Attach this LED to the top of the photodiode (e.g. with a bit of tape or a ring of paper or the 3D printable holder provided) such that whenever the stimulus LED lights up you can clearly see an increase in the input current on the oscilloscope. In addition, configure the 3$^{rd}$ trace on the oscilloscope (blue trace) so that you can see it switch between 0 and 1 to indicate the state of the stimulus.

*[Note, instead of using an LED to self-stimulate via the photodiode, you can cut out the middle man and inject by connecting the cable from port 1 to the analog in port (3), thereby driving Spikeling directly with square pulses. For this crank up dial 2 as well to set the gain (amplitude) of this connection].*

You should now have 3 traces on the screen: The red membrane potential, the green input current, and the blue stimulus. If you cannot see the stimulus, the y-axis is probably configured incorrectly – use the buttons as described before to adjust the scaling of the blue trace. Every time the stimulus is high, the green trace and therefore the red trace should both respond with an increase in turn. This set-up will let you explore the response properties of Spikeling in a more controlled manner.

For now, let's focus on Modes 1 and 2. For both, the stimulus LED switches on and off at regular intervals, such that it is on and off exactly half of the time (the technical term is "**50% duty cycle**"). If you now turn the top-most dial (1) away from resting mid-position, the frequency of this flicker will change accordingly.

**Task 8. Stimulate mode 1 at a constant speed (e.g. 1 Hz) and play with the static input current (dial 4). Can you get Spikeling to reliably spike throughout stimulus presentation, but not spike in the absence of a stimulus? How precisely can you encode the end of the stimulus with spikes?**

*You will probably find that while it is easy to set the neuron to only spike during a stimulus, it is much more difficult to tune it to reliably fire a last spike just before the stimulus ends. This problem will get worse the longer-lasting the stimulus, as the photodiode will adapt just naturally the dropping spike rate. Moreover, any one setting of speed will only "work" with a limited range of settings of static depolarisation. This comes back to the point before – it is much easier to encode the presence of an event with the presence of a spike, than with the absence of an expected spike.*

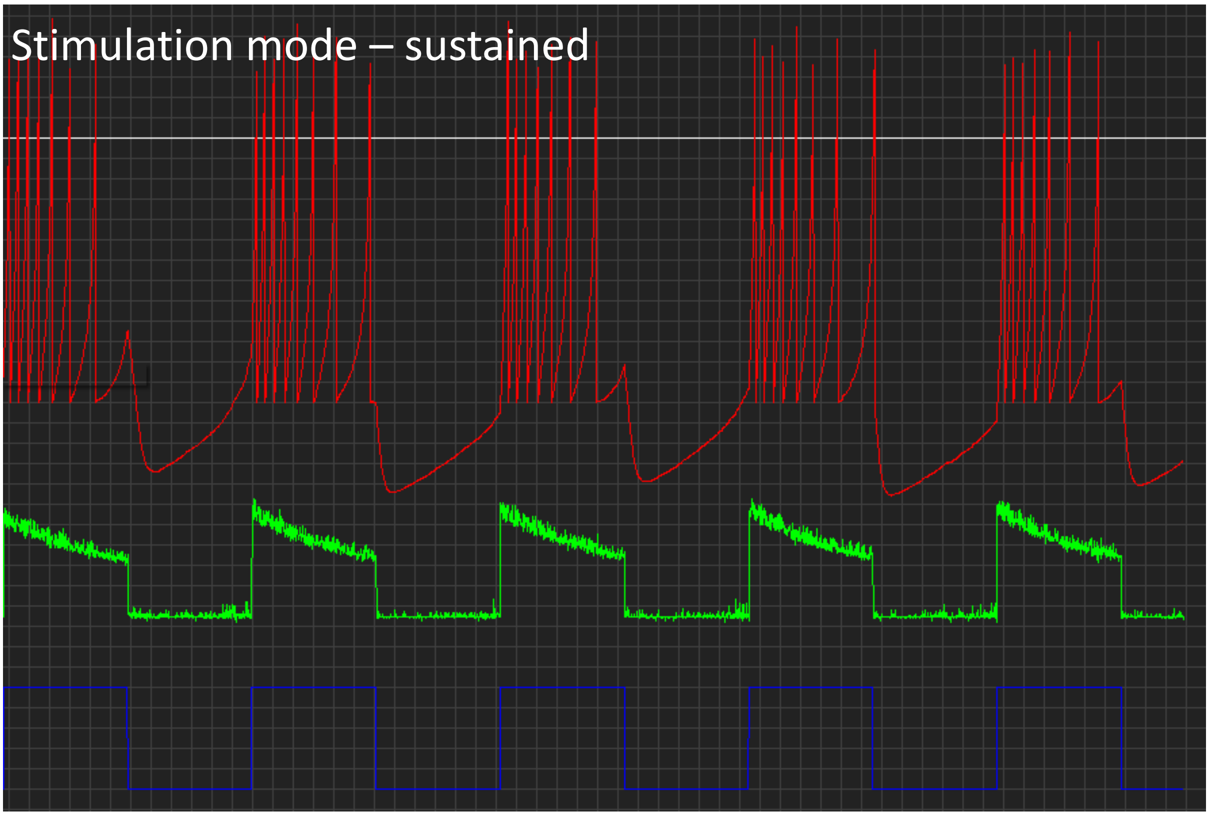


**Task 9. Stimulate mode 2 with steps of light elicited at different speeds. Now, play with the static input current in each mode. Can you get Spikeling to reliably burst during stimulus offset in transient mode 2?**

*You probably found that it is quite easy to set the transient neuron to encode the offset with a burst of spikes, simply because this is an intrinsically bursting neuron. However, depending on the stimulus speed, you may find that this behaviour breaks down quickly. It is however possible to "**entrain**" this neuron (synchronise to the stimulus) using light within a limited range of stimulus rates. As such, as a primary sensory neuron reporting the offset of a step of light, this neuron is not great – as it will also spike simply if you wait long enough assuming it is*

*slightly depolarised. However, if you had an inherently rhythmical stimulus (for example a sensory mechanoreceptor neuron in the leg firing during walking) and you wanted to synchronise the activity of other neurons to this stimulus, such a burst-entrainment can be very useful!*

**Task 11: Now, start playing with the speed of the stimulus while adjusting the static input current as required for all modes. How fast can you go with either the transient or the sustained neurons until you start observing spike failures?**

*You will probably find that all neurons do a reasonable job in encoding each event with at least one spike until about 2-3 Hz, but that at higher frequencies they will start to fail spiking every now and then despite the presence of a stimulus. As Spikeling is set up, it will probably do a little better using the sustained neurons. This is not inevitable – if one were to change the spiking parameters a bit, it would be possible to make the transient neuron more reliable. More importantly, the photodiode, the spike mode settings and the static input current all come into play when determining thefrequency limit at which a neuron can follow a time-varying stimulus. As such,* ***"tuning" a neuron****'s response preference to a desired fluctuating input requires setting a myriad of properties. The nervous system utilises this to tune each neuron to a specific range of input statistics, and, indeed, to adjust these settings as the task at hand requires.*

**Task 12. Pick one mode and keep increasing the speed of the stimulus almost as fast as it will go and set the static input current such that it only spikes occasionally. What do these spikes encode?**

*If you set Spikeling into a regime where most stimuli do not trigger a spike, you will notice that nonetheless each stimulus drives a clear subthreshold response. If you now look carefully at the timing of each spike that is elicited, you will likely find that this time is fairly well "****phase-locked****" to the onset of a stimulus phase. In isolation, this is not a particularly useful property of neurons. But imagine you had 10 such neurons, each spiking unreliably every 5-10 stimulus phases. If you sum the input from these 10 neurons using a larger, postsynaptic neuron, you could fully reconstruct the original stimulus train even though no single input neuron encodes that information. This is called a "****volley code****", and it is one of the most important tricks that neuronal networks use to encode a fast time-varying stimulus that exceeds the speed that any one neuron can encode. For example, in auditory systems, the stimulus frequency can easily reach the kHz range which no neuron can possibly follow, as discussed above. Nonetheless, if you record any one neuron's firing in response to such a stimulus you will probably find that the timing of each spike is exquisitely well phase-locked to the stimulus, such that if you had a few 100 of these neurons you could precisely reconstruct the original input.*

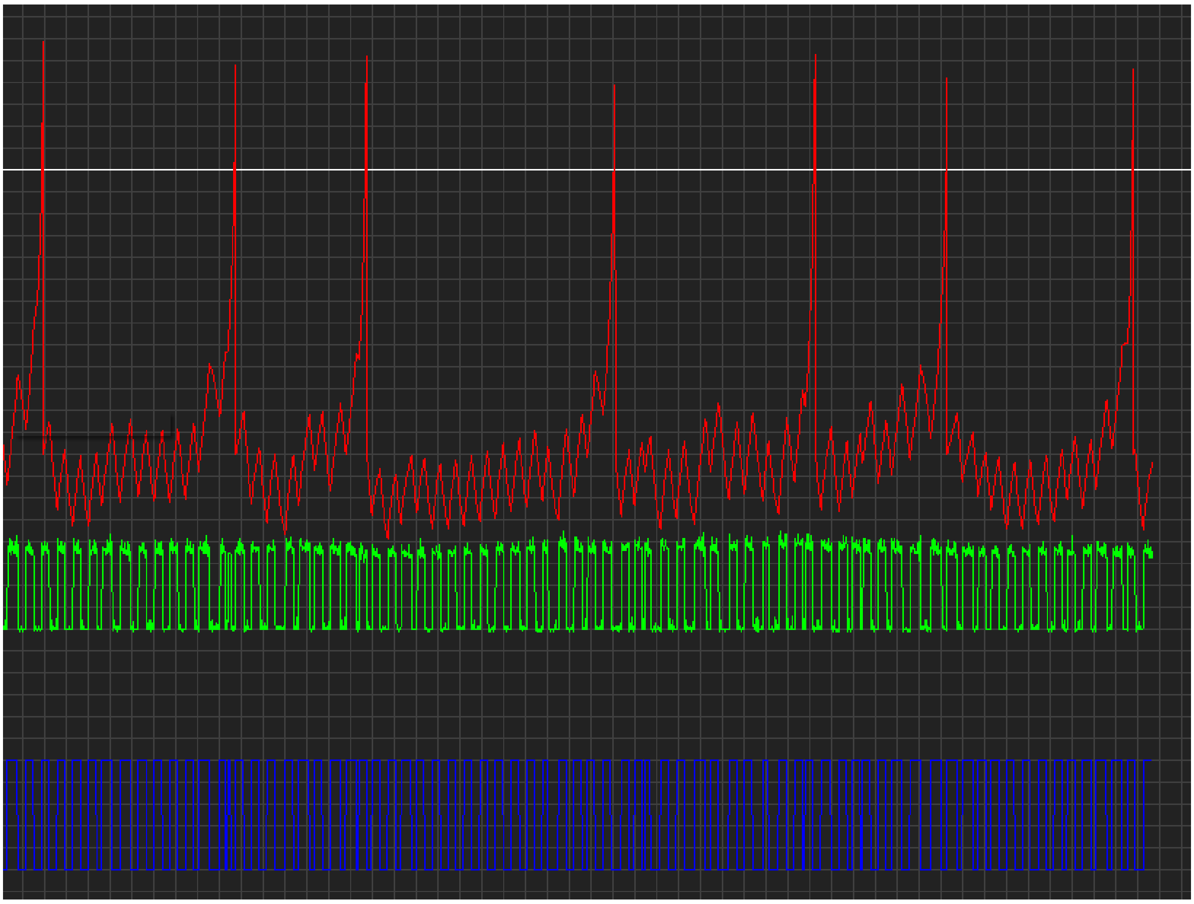

**Task 13. Decrease the static input current until no more spikes are triggered. Observe the membrane potential as you increase the stimulus speed. Can you get Vm to fail following the stimulus?**

*You will probably find that up to the speed that Spikeling can stimulate, Vm comfortably tracks the stimulus (while spikes do not). This is a fundamental property of neurons. The subthreshold response of a neuron is almost always better at following stimuli than the spike response. As discussed above, eliciting a spike and resetting the neuron to be ready to fire the next spike takes time (in Spikeling, this takes >10 ms, some real neurons can reset after 1-2 ms but not faster). If you do not need to generate a spike, this limitation is dramatically reduced, and you can follow stimuli much better, and much more accurately (also in amplitude). Of course,* ***not all neurons spike.*** *Spiking consumes energy and is a relatively costly and inefficient way of conveying information, so if neurons can avoid using spikes, they will. For example, one fundamental reason to use spikes is to rapidly cover large distances. If you have a big brain (like us), you need to use a spike to send a signal from one end to the other. Depending on a neuron's electrotonic properties, a* ***graded signal*** *would just decay with*

*distance and be lost in the noise ~100 microns from the origin. Spikes are regenerative and propagate over distance, allowing signals to be transmitted over longer distances (stubbing your toe). Two disadvantages of signalling with spikes is that you truncate the message, both in time (refractory period) and in amplitude (threshold). So, if the distance to be covered is small, using spikes is usually avoided. For example, the tiny nematode worm C.elegans barely uses any spike at all. The animal is so teeny that all distances are small. Similarly, many neurons in the Drosophila brain don't spike. Other's use a* ***"mix" of spiking and graded processing****. In vertebrates, the same thing happens in some neurons. For example, in the retina, only about half of the neurons use spikes. Photoreceptors, for example, generally don't use spikes. Ganglion cells, on the other hand, connect the eye to the brain via the optic nerve – so there is no way around it, those neurons have to spike.*

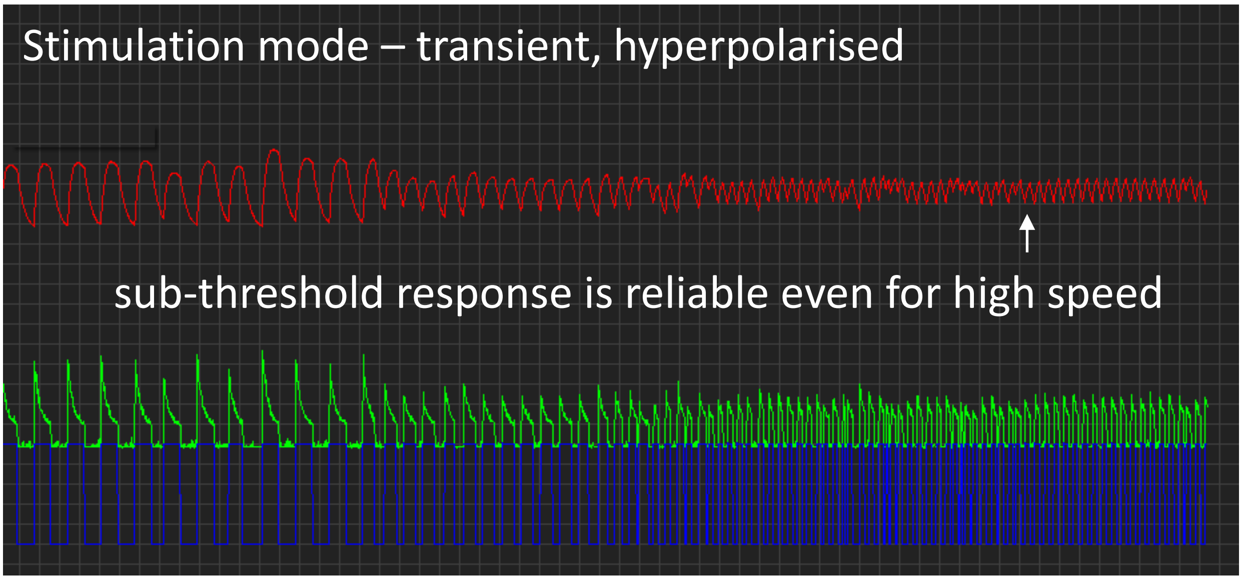


## Noise

Real neurons are noisy. Sources of noise include synaptic inputs, receptor noise, thermal noise or even noise associated with the physical world that an animal inhabits. Accordingly, one major challenge that all neurons face to at least some degree is how to detect the meaningful "**signal**" in the background of meaningless "**noise**". So far, we have been working at low noise but Spikeling provides the option to add different level noise to any operation – for this, turn the second dial from the bottom (dial 3). At mid-point (reset-state) and below that, no noise is added, but above that position increasing the dial will linearly increase a "noisy current" which adds to the total current shown in the green trace.

**Task 14. Turn up the noise dial, while keeping the mean input current roughly centred around 0 (use static input current dial to offset this if necessary). What happens to membrane voltage as you add more and more noise?**

*At first, increasing the noise current will only affect the baseline noise of membrane voltage, which will resemble a* ***low-pass filtered version*** *of the input noise. This is the first "trick" used by all neurons to dampen high frequency components in the noise – the membrane potential simply cannot track the fastest transients in the input current and therefore "automatically"*

*filters them out. As you keep increasing the noise level, you should be able to elicit spikes. These noise-driven spikes are almost always a bad thing. They do not convey any information (thus wasted energy), and to make things worse, they confound any message that the neuron is aiming to encode using spikes. Accordingly, neurons often aim to keep the spike threshold high enough such that the noise they have to deal with by itself very rarely triggers a spike. Accordingly,* ***spike generation can be used as a powerful noise filter****.*

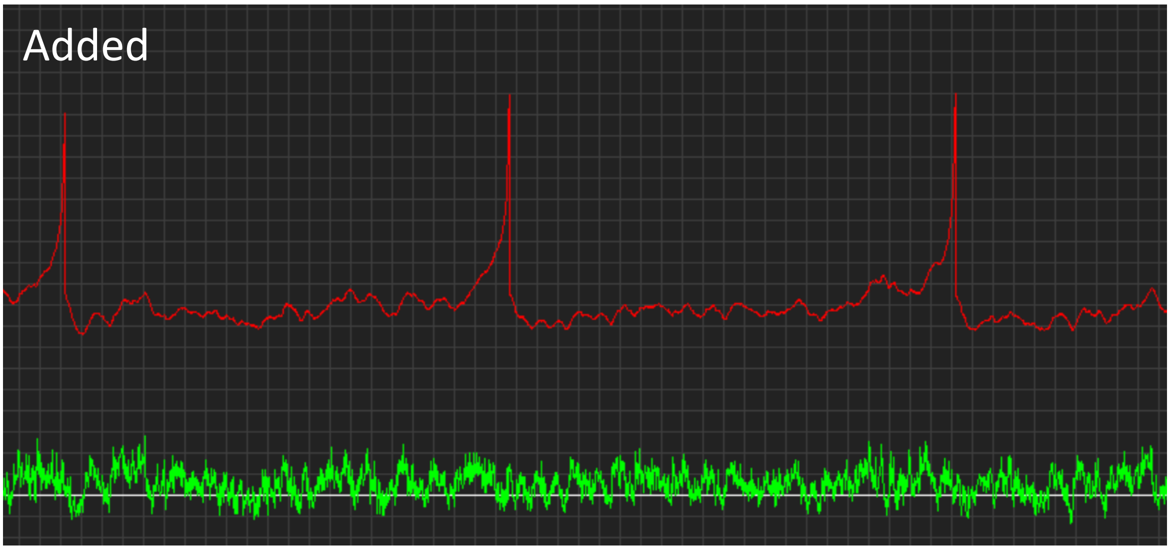


**Task 15. Turn the noise dial (3) back down and look at the baseline. Now cover the photodiode (e.g. with your hand). What happens?**

*You will probably find that when you cover the photodiode, the baseline noise that we had all along in the above exercises decreases. Clearly, the photodiode is introducing noise to the system! In this case this is high frequency noise that largely gets smoothed at the level of membrane potential. All sensory processes, whether electronic as here, or in biology, necessarily introduce noise - simply because the apparatus to pick up the desired physical stimulus is never perfect. In vision, for example, the stimulus (photons) is absorbed inside an opsin-type protein by a chromophore. As the photon arrives, it photoisomerises the chromophore and thereby sets a biochemical cascade into action that ultimately results in the opening of ion channels. However, the isomerisation event can also occur "spontaneously" – or rather driven by heat. As such, all sensory systems are noisy, and nervous systems have evolved a wide range of little tricks to overcome this problem. Here, spike generation or the*

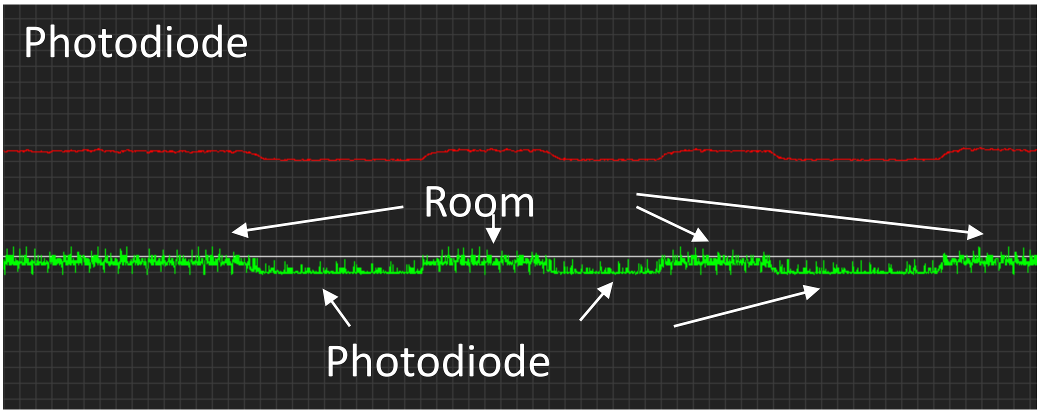


*membrane voltage filtering high frequency noise are but two examples. Others include*

*summation (having multiple neurons signal the same thing and then adding the signal up in a postsynaptic neuron) and temporal "smearing" (e.g. by using slow receptor cascades) are two further examples. There are many more.*

**Task 16. Compare two modes of your choice to see how each deals with noise. Which one would you use to reliably trigger spikes in response to each stimulus? Which one would you choose to make sure spikes are only elicited at the start of a stimulus (even if sometimes they fail)?**

*You will probably find that the sustained neurons will be quite good at reliably firing at least one spike in response to each stimulus. However, the number and timing of each spike per stimulus cycle will likely vary a lot. In contrast, the transient ON neuron might fail every now and then to report the presence of the stimulus, but if there is a spike, it usually means that it was preceded by a stimulus. Spikes from such a neuron are highly informative, but unreliable. How can nervous systems make this mechanism more reliable?*

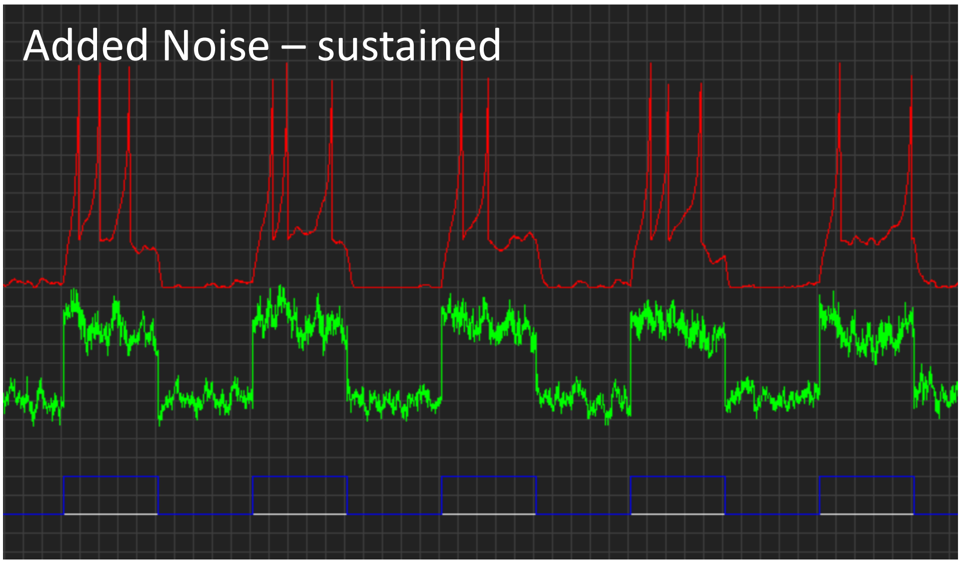

## Building networks

Multiple Spikelings can talk to each other using the Digital Out and Synapse 2 BNC ports (*or Synapse 1, if enabled in the Arduino code, default is disabled*). The idea is that the Digital Out port of one Spikeling (port 4) conveys only spike events, which can be fed into the Synapse 2 input ports of a second Spikeling (port 2). The gain of each synapse can be regulated using the corresponding dial on the other side of the board (dial 2). At reset state, the gain is zero (so the synapse will not do anything if it receives a spike). If you turn the dial up, you get an excitatory synapse. If you turn it below midpoint, you get an inhibitory one.

Try this now: Reset two Spikelings and take a BNC lead to connect the Digital Out port of one (presynaptic) to the Synapse 2 in port of the second. Connect the second Spikeling (postsynaptic) to the PC via the USB cable so that we can read its activity on the oscilloscope. The presynaptic Spikeling does not need to be connected– but it does need to be powered, either using a 9V battery or by plugging the USB into a power socket).

**Task 17. In this configuration (above) increase the static input current on the presynaptic neuron so that it continuously fires a few spikes per second. Now observe what happens to the postsynaptic neuron as you turn up the synapse 2 dial to increase its gain.**

*You should observe that if the dial is at midpoint, nothing much happens – the spikes from the presynaptic Spikeling are still received, but they do not drive any current in the postsynaptic neuron. (Note that you can see the incoming spikes if you open a second oscilloscope – Synapse 2 spikes are on channel 5, so the green channel of the second oscilloscope). Now as you increase the gain you should see that each spike triggers small depolarising current which decays back to baseline within a few 100 ms. If spikes come in a sufficient rate,* ***this input current will start to integrate between successive incoming spikes*** *to further depolarise the cell. If it reaches threshold, spikes should be triggered.* ***The fact that the synaptic current outlasts the duration of the incoming spike is a fundamental ingredient to neuronal processing****. It means neurons can "count and calculate" using synapses – sort*

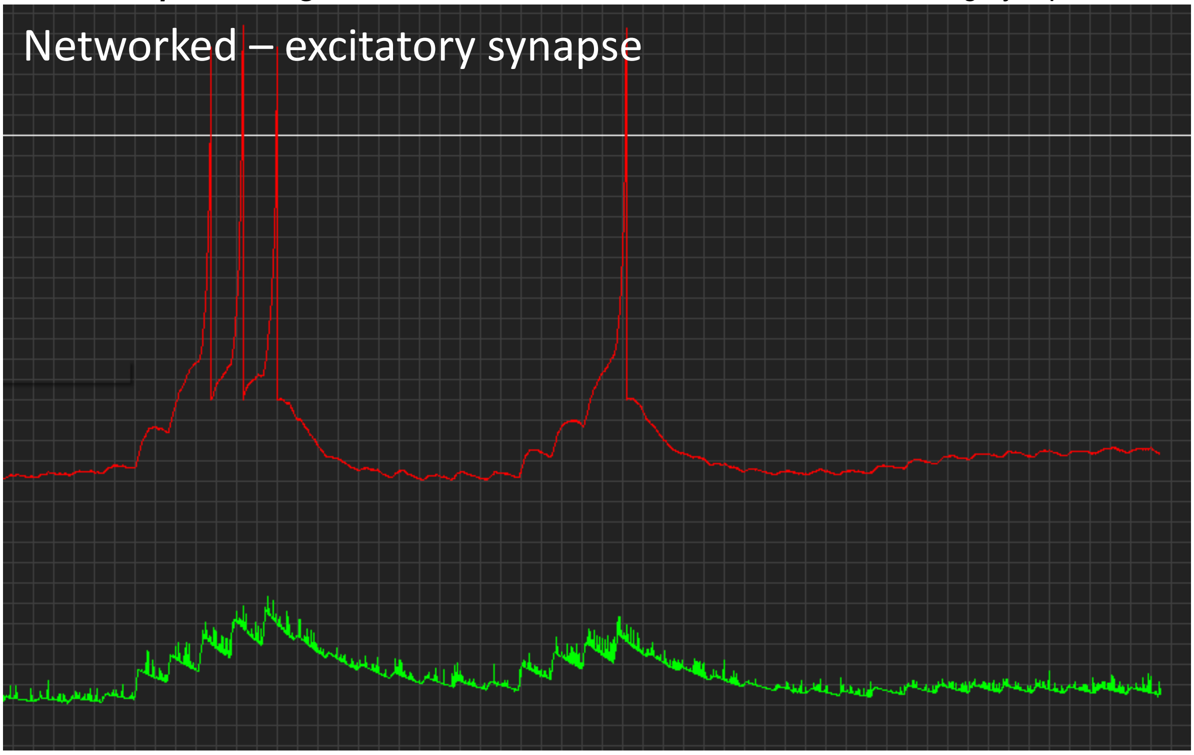

*of, anyway. For example, suppose for a particular computation it is important to know that there was not just one, but two spikes fired within close succession. This could be easily computed by setting the excitatory gain of the connecting synapse(s) such that a single spike does not drive the postsynaptic neuron to threshold, but that if two spikes arrive in close succession, threshold is reached and thus the neuron fires. The same logic also applies across different synapses, which allows the implementation of* ***coincidence detection****.*

**Task 18. Now turn the Synapse 2 dial below midpoint. What happens?**

*You should observe that now each presynaptic spike drives a hyperpolarising current in the postsynaptic neuron. This is intended to mimic an inhibitory connection. In case of Spikeling, this connection is programmed to be mirror symmetric to the excitatory connection in every way. However, in reality the gain and time courses of synaptic events can vary dramatically and are another fundamental ingredient to building computational networks. For the purpose of this tutorial, the time-course of the synapses is fixed. However, advanced users can change this in the annotated Arduino code.*

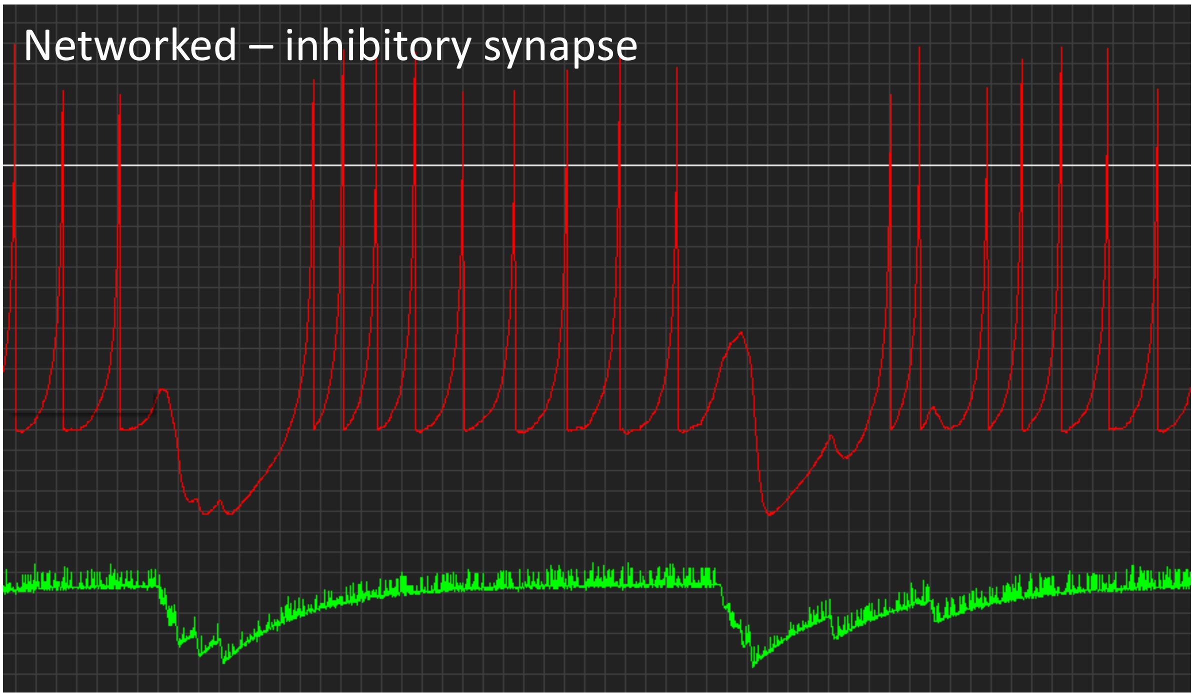


**Task 19. Put the postsynaptic neuron's stimulus LED above the photodiode as before, but now hyperpolarise it using the static input dial such that the LED alone does not elicit spikes. On the presynaptic neuron, make sure that it does not fire spikes at rest, and configure its input to be weakly excitatory. Now take a torch to stimulate the photodiode on the presynaptic neuron and thereby make it spike. Can you get the postsynaptic neuron to spike?**

*Neither the stimulus light nor the synaptic excitation alone should be sufficient to drive a spike in the postsynaptic neuron. However, if you make the presynaptic neuron spike using the torch at the same time as the stimulus comes on, the two excitatory inputs will summate and you should be able to reach threshold. This is an example of a* ***coincidence detector****. If the*

*postsynaptic neuron spikes, it means that both the stimulus (stimulator) AND the presynaptic stimulus (torch) were both active at the same time.*

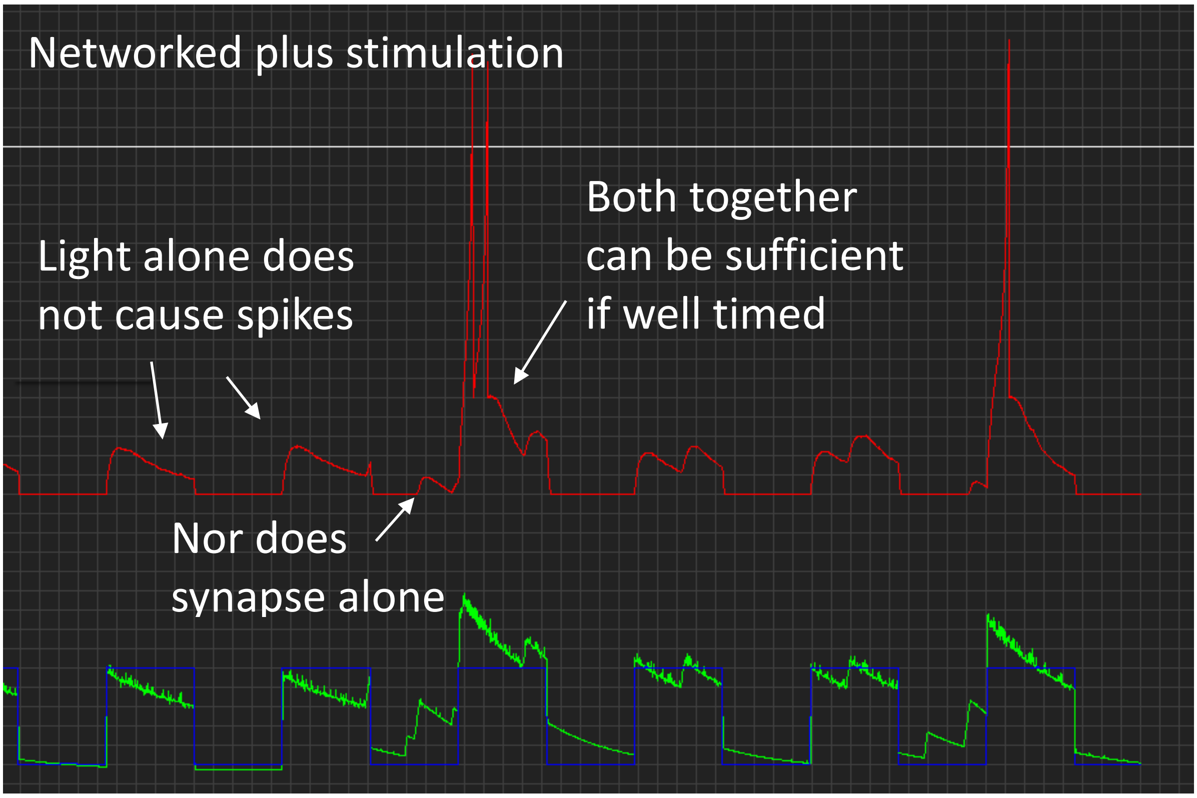


*One "famous" coincidence detector is used in auditory systems for comparing the signal between two ears. Imagine sound coming from your left. Because sound travels slowly (330 m/s) it will arrive at your left earhole about a millisecond before it arrives at your right earhole. Neurons in each ear's cochlea will spike as soon as the sound arrives and send that signal to the brainstem where the signals from the two ears are combined. Here there are bilateral neurons that receive inputs from both ears. These are the coincidence detectors. If the sound came from the left, the spike from the left ear comes in before the spike from the right ear and its associated postsynaptic current will decay rapidly, such that it does not overlap with the current triggered by a spike from the right ear. As a result, the central neuron will not spike. However, if the sound comes from e.g. straight ahead, it will reach both ears are the same time which in turn means that the left and right spike inputs to the central neuron will coincide and drive a postsynaptic spike. Such as central neuron therefore encodes the direction of sound. If we now slightly offset the speed by which the spikes travel, or implement delays of initial spike generation in either ear, we can build a neuron that is selective for any sound direction in the azimuth plane (horizontally around the head).*

*This kind of logic expands into a multitude of computational operations. The above coincidence detector is perhaps the simplest of* ***logical operations****: an "AND" gate. This means that Input 1 AND Input 2 need to be "high" for the spike to be triggered. Another logical operation is an "OR" gate – Input 1 OR Input 2 need to be high. This is easily achieved by increasing the synaptic gain such that one neuron alone can drive the postsynaptic spike. There are many such logical operations (e.g. NOR, XOR, XNOR), which can be thought of a computational building blocks of electronics and neuronal networks alike. If you simulate many neurons in a computer, it is straightforward to implement any of these logical gates. We can*

*try to set-up some of these using Spikeling. For example, how might you build an "inverse conditional" – i.e. a neuron that only fires if input 1 is high, unless input 2 is also high? How might you build a NOR gate (only fires when both Inputs 1 and 2 are "low"). How might you build an NAND gate (fires always except when both 1 and 2 are "high"). If you have more than two Spikelings at hand, try playing with these a bit. What kind of computations can you implement? (one useful reference for logical gates and their implementation and background can be found on Wikipedia: [https://en.wikipedia.org/wiki/Logical_connective](https://en.wikipedia.org/wiki/Logical_connective)).*

*Note that in their "pure form", logical operations are Boolean ("True or False, no intermediate state is possible"). But neurons are noisy, so not all operations are equally "easy" to implement! (all are possible though).*

**Task 20. Using what you have learnt(!) (and two Spikelings): Can you build an "OFF neuron" using only ON neurons as the input?**

*One way of doing this is by setting the postsynaptic neuron at a high resting potential such that it continuously generates spikes. Now take a presynaptic neuron that responds to the light in an "On fashion" and connect it to inhibit the continuously active cell. If you tune the inputs right, you should be able to generate something like the below:*

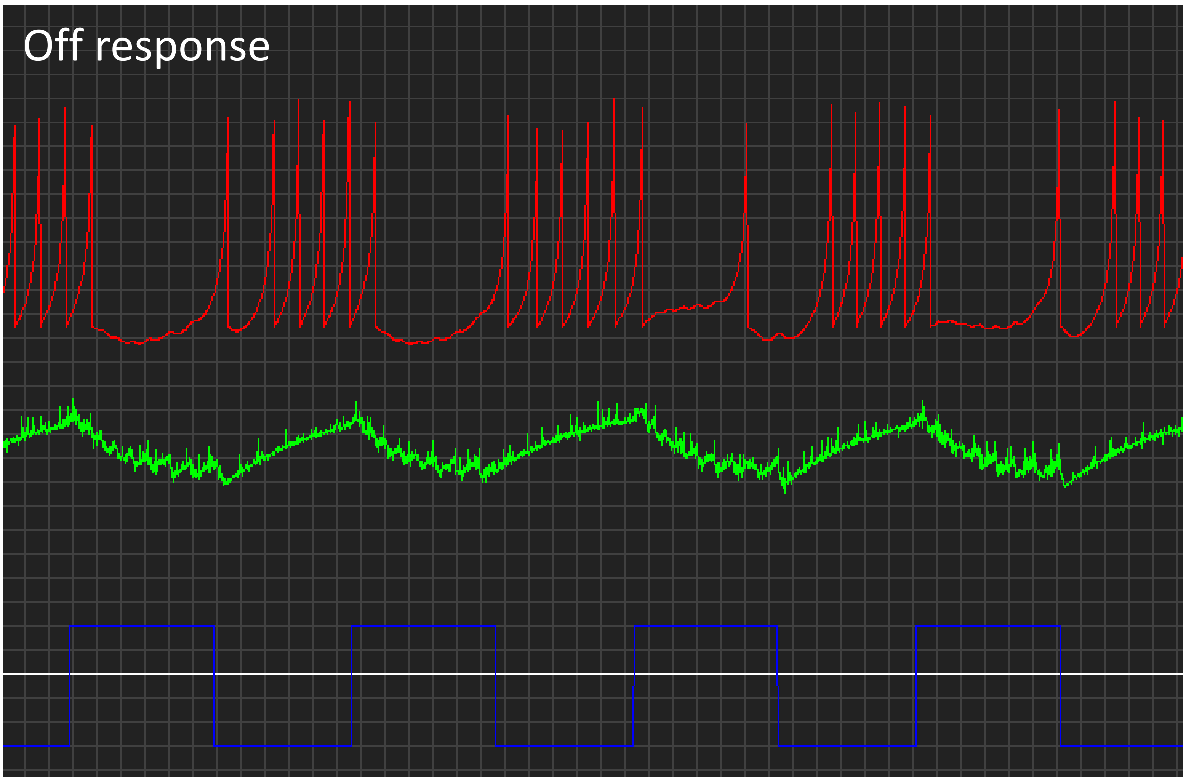


*This is not a very "good" Off cell. It is a bad Off cell. Sometimes it spikes during the light, and more problematically, the timing of the 1st spike after the light switches off is very variable. This is because now we are not using the presence of an excitatory drive, but rather the absence of an inhibitory drive as signal to generate spikes. Using this setup, you will find it very difficult indeed to tune the off cell to become more transient. The problem is that the synaptic current of the inhibition is slow to decay so after the stimulus switches off the inhibition will take a while to settle back down to zero. This results in a slow upwards trend of the membrane voltage which, as we saw in the beginning, is a very ineffective way to trigger*

*spikes. So, to make it a "better" Off cell, we would need to implement something that makes the membrane potential increase more rapidly after the inhibition turns off. One way to achieve this would be the implementation of a "**rebound spike**, while another would be the use of a graded (non-spiking) network feeding into Spikeling (as we saw before, membrane voltage is much better at tracking fast inputs below spike-threshold). While Spikeling could principally be programmed to mimic either or both of these possibilities, we will not be covering them here.*

**Task 21. Using what you have learnt (and two Spikelings), can you build a "delayed ON neuron"? This is a neuron that responds with spike(s) after the onset of a stimulus, but with a delay?**

*One way of implementing this delayed ON cell would be to use an excitatory synapse that requires more than one presynaptic spike to trigger a postsynaptic spike as shown below. Note the two separate small depolarising current that precede each other by ~100 ms – these come from the presynaptic neuron responding to the light with 2 spikes in close succession. The second incoming spike takes the postsynaptic neuron past threshold.*

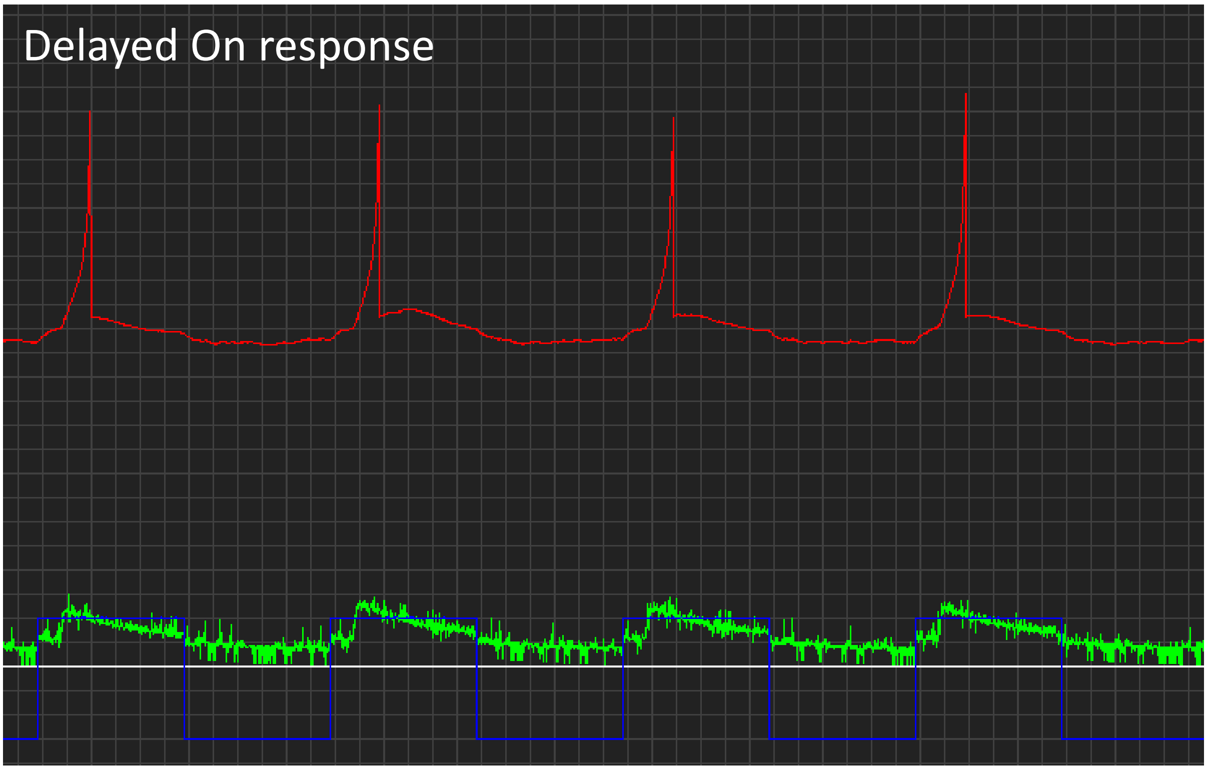


**Task 22 (final!):**

**Using the delayed ON neuron circuit, plus an additional non-delayed ON transient neuron, can you build an elementary motion detector? (Hint: This will probably require 3 neurons!)**
